# Inhibition of mitochondrial respiration prevents *BRAF*-mutant melanoma brain metastasis

**DOI:** 10.1186/s40478-019-0712-8

**Published:** 2019-04-10

**Authors:** Terje Sundstrøm, Lars Prestegarden, Francisco Azuaje, Synnøve Nymark Aasen, Gro Vatne Røsland, Jobin K. Varughese, Marzieh Bahador, Simon Bernatz, Yannick Braun, Patrick N. Harter, Kai Ove Skaftnesmo, Elizabeth S. Ingham, Lisa M. Mahakian, Sarah Tam, Clifford G. Tepper, Kjell Petersen, Katherine W. Ferrara, Karl Johan Tronstad, Morten Lund-Johansen, Rudi Beschorner, Rolf Bjerkvig, Frits Thorsen

**Affiliations:** 1 Kristian Gerhard Jebsen Brain Tumour Research Centre, Department of Biomedicine, University of Bergen, Jonas Lies vei 91, 5009 Bergen, Norway; 2 Department of Clinical Medicine, University of Bergen, Haukelandsveien 22, 5021 Bergen, Norway; 3 Department of Neurosurgery, Haukeland University Hospital, Haukelandsveien 22, 5021 Bergen, Norway; 4 Department of Dermatology, Haukeland University Hospital, Haukelandsveien 22, 5021 Bergen, Norway; 5 NorLux Neuro-Oncology Laboratory, Department of Oncology, Luxembourg Institute of Health, 84 Val Fleuri, 1526 Luxembourg, Luxembourg; 6 Present address: Proteome and Genome Research Unit, Department of Oncology, Luxembourg Institute of Health, 1A-B, rue Thomas Edison, L-1445 Strassen, Luxembourg Luxembourg; 7 Department of Oncology and Medical Physics, Haukeland University Hospital, Haukelandsveien 22, 5021 Bergen, Norway; 8 Department of Biomedicine, University of Bergen, Jonas Lies vei 91, 5009 Bergen, Norway; 9 Edinger-Institute (Neurological Institute), Goethe-University Medical School, Heinrich-Hoffmann-Strasse 7, 60528 Frankfurt am Main, Germany; 10 Institute of Marine Research, Nordnesgaten 50, 5005 Bergen, Norway; 11 Department of Biomedical Engineering, University of California Davis, 451 East Health Sciences Drive, Davis, CA 95616 USA; 12 Department of Biochemistry and Molecular Medicine, UC Davis Comprehensive Cancer Center, 4645 Second Avenue, Sacramento, CA 95817 USA; 13 Computational Biology Unit, Department of Informatics, University of Bergen, Thormøhlensgate 55, 5008 Bergen, Norway; 14 Institute of Pathology and Neuropathology, Department of Neuropathology, University of Tuebingen, Tuebingen, Germany; 15 The Molecular Imaging Center, Department of Biomedicine, University of Bergen, Jonas Lies vei 91, 5009 Bergen, Norway

**Keywords:** Cancer, Melanoma, Brain metastasis, BRAF V600E, β-Sitosterol, Treatment

## Abstract

Melanoma patients carry a high risk of developing brain metastases, and improvements in survival are still measured in weeks or months. Durable disease control within the brain is impeded by poor drug penetration across the blood-brain barrier, as well as intrinsic and acquired drug resistance. Augmented mitochondrial respiration is a key resistance mechanism in *BRAF*-mutant melanomas but, as we show in this study, this dependence on mitochondrial respiration may also be exploited therapeutically. We first used high-throughput pharmacogenomic profiling to identify potentially repurposable compounds against *BRAF*-mutant melanoma brain metastases. One of the compounds identified was β-sitosterol, a well-tolerated and brain-penetrable phytosterol. Here we show that β-sitosterol attenuates melanoma cell growth in vitro and also inhibits brain metastasis formation in vivo. Functional analyses indicated that the therapeutic potential of β-sitosterol was linked to mitochondrial interference. Mechanistically, β-sitosterol effectively reduced mitochondrial respiratory capacity, mediated by an inhibition of mitochondrial complex I. The net result of this action was increased oxidative stress that led to apoptosis. This effect was only seen in tumor cells, and not in normal cells. Large-scale analyses of human melanoma brain metastases indicated a significant role of mitochondrial complex I compared to brain metastases from other cancers. Finally, we observed completely abrogated BRAF inhibitor resistance when vemurafenib was combined with either β-sitosterol or a functional knockdown of mitochondrial complex I. In conclusion, based on its favorable tolerability, excellent brain bioavailability, and capacity to inhibit mitochondrial respiration, β-sitosterol represents a promising adjuvant to BRAF inhibitor therapy in patients with, or at risk for, melanoma brain metastases.

**Electronic supplementary material:**

The online version of this article (10.1186/s40478-019-0712-8) contains supplementary material, which is available to authorized users.

## Introduction

Melanoma incidence rates are increasing, and brain metastases represent a leading cause of melanoma-associated deaths [10, 14, 17, 48]. Even though phase II clinical trials have shown promising therapeutic effects by the use of BRAF inhibitors (BRAFi) + MEK inhibitors (MEKi), resistance still develops [18, 32]. Also, in experimental studies it has been shown that immunotherapy combining anti-PD1 plus CTLA4 may have a therapeutic effect [59]. However, a major problem is the presence of the blood-brain barrier (BBB), which is intact during early stages of brain metastasis development [51]. Although the presence of metastases can compromise the structure and integrity of the BBB, it is still a significant obstacle for efficient drug delivery [22, 31, 37, 66]. Moreover, cancer cells that have extravasated to the brain parenchyma may find protection within the brain microenvironment, and be more prone to develop resistance due to sub-therapeutic drug concentrations [46, 52]. There are also significant concerns associated with drug-related adverse effects, patient selection, and costs versus benefits [56, 67]. Thus, there is a prevailing need to find new therapeutic and preventive approaches that offer improved and sustained responses against brain metastases [3, 52].

Recently, it has been shown that molecular drivers of cellular metabolic reprogramming events may be critical in tumor development, metastasis formation and drug resistance [1]. For instance, subsets of melanomas that show primary resistance to targeted therapies seem to rely more on mitochondrial respiration than glycolysis [45, 63]. Furthermore, when *BRAF*-mutant melanomas are treated with vemurafenib (a BRAFi), the MITF-PGC1α axis is up-regulated, which leads to increased mitochondrial respiration and scavenging of reactive oxygen species (ROS) [24]. These intrinsic and acquired resistance mechanisms provide obvious survival advantages, but the dependence on mitochondrial respiration may also be exploited therapeutically [24, 45, 49, 63, 69].

The metabolic alterations that occur in melanoma brain metastases are largely unknown, but cell lines derived from metastatic melanomas and melanoma metastases (non-brain) have shown elevated levels of mitochondrial respiration when compared to primary melanomas [7, 26]. In the brain, metastatic breast cancer cells have been shown to be less dependent on glucose, and instead utilize mitochondrial respiration for energy production and antioxidant defense [15, 16]. Moreover, metastatic breast cancer cells have been shown to display neuron-like characteristics in the brain microenvironment [39, 41]. Whether these changes reflect intrinsic or adaptive capabilities of tumor cells to thrive in the neural niche remains to be determined. Nevertheless, to survive and grow, extravasated cancer cells can adjust to the lower glucose levels in the brain interstitium [16]. Notably, when cancer cells are deprived of glucose they switch from glycolysis to mitochondrial respiration and become sensitive to low doses of mitochondrial complex I (CI) inhibitors that do not affect normal (non-cancerous) cells [40].

In this study (scientific flowchart outlined in Additional file 1: Figure S1), using a well-established patient derived metastatic xenograft model, we identified a melanoma brain metastasis gene signature by RNA-sequencing (RNA-seq). We then utilized the Connectivity Map drug discovery tool (cMap; Broad Institute) to search for putative therapeutic compounds with the potential to invert the signature. The lead compounds identified were then tested in predictive xenograft models of *BRAF*-mutant melanoma brain metastases. Here, we show that β-sitosterol, a well-tolerated and brain-penetrable phytosterol, effectively prevented the emergence of brain metastases leading to a significant survival benefit. Mechanistically, we show that β-sitosterol targets mitochondrial CI that leads to an inhibition of mitochondrial respiration. We also show that β-sitosterol abrogates potential resistance to BRAFi, inferring a clear therapeutic rationale for using β-sitosterol as a therapeutic agent towards melanoma metastases.

## Materials and methods

### Cell lines

The H1 cell line was generated from a human melanoma brain metastasis and transduced with lentiviral vectors expressing the genes for GFP and luciferase (H1_DL2) as previously described [60], or shRNAs targeting NDUFA8 (H1_shNDUFA8; TAGAAGACGCACCGGCGGTGTTTAGGGGAAGGTAAAGTTAATATTCATAGCTTTGCCTTCCTCTAAACACCGTTTTTTGGCAAGCAAAAGACGGCATACGAGATATGTACCAGTCAGTACCAGTTTCGCCGTCTTCGT). The Melmet 1 and Melmet 5 melanoma cell lines were developed from a skin and a lymph node metastasis, respectively, and were kindly provided by Ø. Fodstad (University of Oslo, Oslo, Norway). The A375 cell line was purchased from the American Type Culture Collection (ATCC). A human lung fibroblast cell line, SV-80, was purchased from CLS Cell Lines Service GmbH. The PC14-PE6 cell line was kindly provided by F. Winkler (University Hospital Heidelberg & German Cancer Research Center, Heidelberg, Germany), and a brain seeking subline PC14_PE6_Br2 was generated in our laboratory as described earlier [27]. The Melmet 1 and Melmet 5 cell lines were transduced with a lentiviral pGF1-CMV reporter vector that co-expresses copGFP and firefly luciferase linked by the self-cleaving peptide T2A (System Biosciences). The immortalized human melanocytes-hTERT (cat. no. T0463) and astrocytes (cat.no. T0281) were both purchased from Applied Biological Materials (Richmond, BC, Canada). The primary epidermal melanocytes HEMa (cat.no. PCS-200-013) were purchased from ATCC. The H1, Melmet 1, Melmet 5 and A375 cell lines have the *BRAF*^*V600E*^ mutation. The transduced cell lines were used in all experiments, authenticated within the last six months using short tandem repeat (STR) profiling, and maintained as previously described [57].

### In vivo generation of samples for RNA sequencing

Eight weeks old female NOD.CB17-*Prkdc*^*scid*^/NcrCrl mice were purchased from Charles River Laboratories International. Anesthesia was induced with 3% and maintained with 1.5% isofluorane in oxygen. We performed intracardiac injections in seven mice (5 × 10^5^ H1_DL2 cells suspended in 0.1 mL PBS) as previously described [60]. Whole-body bioluminescence imaging (BLI) was used to evaluate injection failure 10 min post-injection and weekly over seven weeks to monitor metastasis formation (Additional file 2: Figure S2a) using a Xenogen Ivis 100 Small Animal Molecular Imager (Xenogen Corporation) as previously described [60]. At seven weeks, we injected 150 mg/kg D-luciferin Firefly (Gold Biotechnology), sacrificed the mice ten min later, and performed ex vivo BLI to evaluate organ involvement in detail. All animals invariably developed metastases in their brain, adrenals, ovaries, and femurs, and these organs from five animals showing the highest tumor burden on BLI, were dissociated using tailored protocols based on the Liberase TM Research Grade enzyme reagent (Roche Applied Science; Additional file 3 Table S1). Sample yields of GFP positive tumor cells were checked by fluorescence microscopy prior to cell sorting (Additional file 2: Figure S2b) using the BD Influx high-speed cell sorter (BD Biosciences). We aimed for a minimum of 150,000 cells in each sample, and picked the three tumor cell samples from each organ with the most cells for subsequent RNA sequencing: 1) brain (151,023, 150,835 and 154,797 cells); 2) adrenals (151,968, 184,506 and 276,146 cells); 3) ovaries (159,736, 171,814 and 433,703 cells); and 4) femurs (120,530, 91,055 and 73,942 cells). These 12 samples (3 samples × 4 organs) of metastatic melanoma cells were kept in 1% FBS medium, pelleted by centrifugation, transported on dry ice and stored in a − 80 °C freezer.

### RNA isolation

Total cellular RNA was isolated from the cell pellets using the TRIzol reagent (Life Technologies) and a modified protocol that incorporates an additional extraction with phenol/chloroform/isoamyl alcohol (25:24:1, pH 4.3). RNA quantity and quality were assessed on a NanoDrop spectrophotometer (Thermo Scientific) and the Agilent 2100 Bioanalyzer (Agilent Technologies), respectively.

### Library preparation for RNA sequencing

RNA sequencing (RNA-Seq) libraries were prepared from 1.0 μg total RNA using the TruSeq RNA Sample Preparation Kit (Illumina) according to the manufacturer’s protocol. Briefly, poly-adenylated mRNA was purified from total RNA and ribosomal RNA removed by two rounds of binding to magnetic poly-dT beads. This was followed by RNA fragmentation by incubation in the presence of divalent cations at 94 °C for 5 min. Double-stranded cDNA was then generated by random-primed first-strand synthesis with SuperScript II reverse transcriptase and subsequent second strand synthesis with RNase H and DNA Polymerase I. The cDNA was then blunt-ended with T4 and Klenow DNA polymerases to remove the 3′-overhangs and fill in 5′-overhangs, phosphorylated with T4 PNK, and then 3′-A tailed by incubation with Klenow Fragment (3′ → 5′ exo–) and dATP. Illumina paired-end (PE) adapters were then ligated, followed by purification with AMPure XP beads. The library was then enriched by high-fidelity PCR amplification (15 cycles) with Phusion DNA Polymerase (Finnzymes Oy) and adapter-specific primers. The molar concentration of the libraries was determined by measuring concentration with a Qubit fluorometer (Invitrogen), determining the insert length with an Agilent 2100 Bioanalyzer, and then qPCR-based quantification (KAPA Library Quantification Kit).

### RNA sequencing

Indexed libraries were pooled, loaded on TruSeq paired-end flow cells, and paired-end sequencing (2 × 100 bp, paired-end; 4 libraries/lane) was performed with an Illumina HiSeq 2000 sequencing system (BGI@UC Davis Joint Genome Center) using standard Illumina kitted reagents (TruSeq SBS Kit v3-HS). Image processing, base calling, quality scoring (Phred), and sample demultiplexing were executed by HiSeq Control Software with Real Time Analysis (HCS 1.5/RTA 1.13) and CASAVA 1.8 software (Illumina). The Tuxedo protocol (http://compbio.mit.edu/cummeRbund) was followed using the alternate protocol of quantification of reference annotation only. We used Ensembl’s human genome build 19, and supplied TopHat with a set of known transcripts from Ensembl. The three BAM files from each individual organ were merged using Picard 1.91 (http://broadinstitute.github.io/picard), and these merged files were subsequently visualized using Integrative Genomics Viewer 2.3 (http://www.broadinstitute.org/igv). The cummeRbund package (http://compbio.mit.edu/cummeRbund) and Partek Genomics Suite 6.12 (Partek Inc.) were used to produce visualizations (Additional file 2: Figure S2c).

### Gene expression analysis and brain metastasis gene signature

**Fig. 1:**
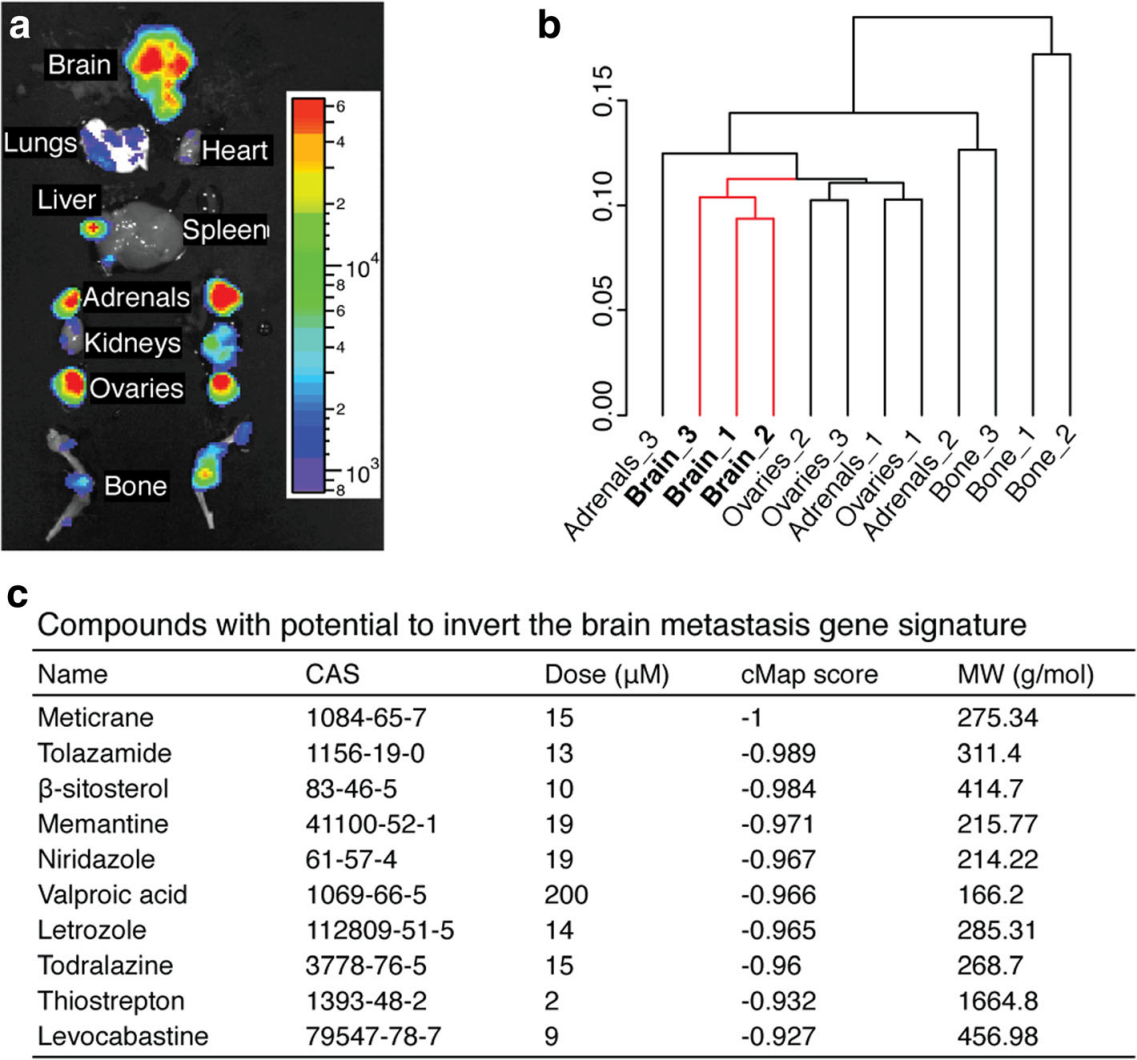
Prediction of candidate compounds from inverse gene profiling. **a** Detailed mapping of organ involvement by ex vivo BLI of a representative NOD.CB17-*Prkdc*^*scid*^/NcrCrl mouse seven weeks after intracardial injection of 5 × 10^5^ H1_DL2 cells (*n* = 7). **b** Dendrogram of all replicates (*n* = 3 per organ) based upon cluster analysis of gene expression profiles. Numbers 1, 2 and 3 indicate sample ID. **c** Top ten compounds from the Connectivity Map (cMap) analysis with the potential to induce the opposite transcriptional response to the brain metastasis signature («anti-brain metastasis signature»). Chemical abstracts service (CAS) registry numbers, reported dose tested, cMap score and molecular weight (M_W_) are provided. A cMap score of − 1 indicates complete reversal of the signature (negative correlation)

We developed an integrated workflow of several independent analyses to build a brain metastasis gene expression profile (Fig. 1b and Additional file 2: Figure S2d). First, there were 134 genes differentially expressed in brain metastases and metastases to any of the other organs; 122 genes were upregulated and 54 were downregulated in brain (some genes appeared in multiple lists). Second, we used Prediction Analysis for Microarrays (PAM; http://statweb.stanford.edu/~tibs/PAM) to identify which of the 134 genes consistently distinguished brain metastases from other organ metastases. We found ten upregulated genes specific to brain metastases. Third, we validated eight of these ten genes using a combination of other computational analyses: A supervised rank product analysis (RankProd; http://www.bioconductor.org/packages/release/bioc/html/RankProd.html) of brain metastases compared with all the other organs pooled together (pfp < 0.01); a meta-analysis of rank product analyses (RankProd) of brain metastases versus each of the other organs (pfp < 0.01); and significance analysis of microarrays (http://statweb.stanford.edu/~tibs/SAM) with a q-value cutoff of 0.05. Fourth, we performed hierarchical clustering using J-Express 2012 (http://jexpress.bioinfo.no/site) and identified 46 genes that clustered together with the cross-validated eight-gene list; these 54 up-regulated genes were used to build a brain metastasis gene signature. Fifth, to enable analysis with the Connectivity Map (cMap) database, we appended the 54 most downregulated genes from the supervised rank product analysis (RankProd) of brain metastases when compared with all the other organs pooled together; all of these 54 downregulated genes were also featured in the 134-gene list of differentially expressed genes, as described above. Thus, the 108-gene brain metastasis signature was comprised of 54 upregulated and 54 downregulated genes (Additional file 2: Figure S2d).

### Computational prediction of candidate compounds

We queried the cMap build 2 database (http://www.broadinstitute.org/cmap) using our 108-gene signature for candidate drugs. cMap is an initiative by the Broad Institute of MIT and Harvard, where the effects of 1309 small molecules on a number of cultured human cells were examined systematically, resulting in a collection of more than 7000 expression profiles that can be freely queried using their web interface. This resource can be used to select for drugs that produce a disease-negating gene signature. In this study, cMap was hypothesized to predict drugs with the potential to induce opposite expression profiles to those observed in our brain metastasis gene signature (Additional file 2: Figure S2e). Before cMap analysis, gene symbols were mapped to HG-U133A probe IDs (microarray used in cMap). The HG-U133A probe file (Platform GPL96) was downloaded and mapping was done with a code written in our laboratory. Of the 54 upregulated genes, 37 genes had at least one matching probe and 55 probes were retrieved in total. Of the 54 downregulated genes, 29 genes had at least one matching probe, and 40 probes were retrieved in total. Mappings were subsequently verified with the GeneAnnot (http://genecards.weizmann.ac.il/geneannot/index.shtml) and GeneCards (http://www.genecards.org) databases.

### Candidate drugs

Nine drugs were purchased from Santa Cruz Biotechnology, Inc.: Meticrane, tolazamide, β-sitosterol, memantine hydrochloride (herein referred to as memantine), valproic acid, letrozole, todralazine, thiostrepton, and levocabastine. Niridazole was not available. For in vitro screening, all compounds were dissolved in 100% dimethyl sulfoxide (DMSO) to a stock concentration of 250 mM. All stock solutions were subsequently diluted in complete DMEM to nine working solutions ranging from 0.2 to 1312.3 μM. This dose spectrum covered well below and above the reported dose levels for all drugs described in cMap. PLX4720 (ChemieTek) was solubilized and diluted in a similar manner to nine working solutions ranging from 0.01 to 1562.5 μM. Prior to in vitro testing, we pre-warmed (37 °C) and sonicated all working solutions.

### Proliferation assays

**Fig. 2:**
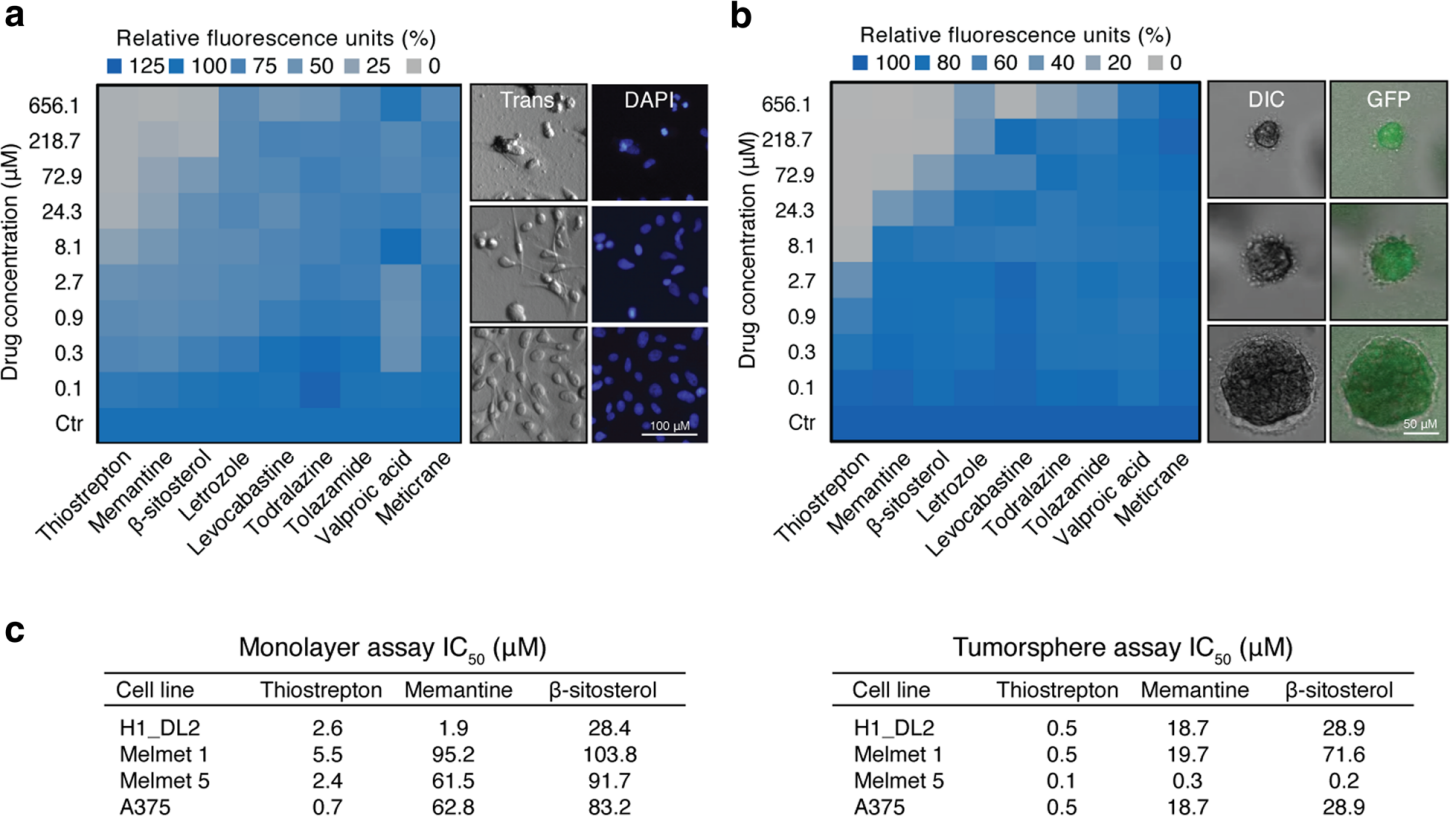
In vitro drug screening. **a** Heatmap of three days H1_DL2 monolayer proliferation assays (left panel) and representative transmission (Trans) and 4′,6-Diamidino-2-Phenylindole (DAPI) images (right panel). **b** Heatmap of ten days H1_DL2 tumorsphere assays (left panel) and representative differential interference contrast (DIC) and green fluorescent protein (GFP) fluorescence microscopy images (right panel). **a-b** Right image panels show examples of β-sitosterol 218.7 μM (top), β-sitosterol 2.7 μM (middle) and control (bottom). **c** Half maximal inhibitory concentration (IC_50_) values for the three most potent compounds across different cell lines and assays. (a–c) Mean; *n* = 6 per cell line per drug per drug concentration. See Additional file 4: Figure S3a–c for more details on this experiment

Cells from four BRAF mutated cell lines (H1_DL2, Melmet 1, Melmet 5, A375) and three normal cell lines (SV80; fibroblasts, hTERT melanocytes and human astrocytes) were quantified using a hemocytometer and seeded into 96-well plates (5000 cells per well) in 100 μL complete DMEM. After 24 h incubation, we added 100 μL of the nine graded candidate drug dilutions, PLX4720, or 100 μL 0.1% or 1.0% DMSO in complete DMEM to each well (*n* = 18 per cell line per drug per drug concentration). Three days later, we added 20 μL of resazurin 0.1 mg/mL (Sigma-Aldrich Co.) per well and read the plates 4 h later (Fig. 2a and Additional file 4: Figure S3a-f) using a VICTOR X3 multilabel plate reader (PerkinElmer) with Workout 2.5 data analysis software (560 nm excitation and 590 nm emission). Wells with 200 μL complete DMEM and no cells were used for background correction (*n* = 60). IC_50_ values were calculated (Fig. 2c, and Additional file 4: Figure S3) using GraphPad Prism 6 for Mac OS X (GraphPad Software Inc.).

Cells were fixed by adding 50 μL 8% paraformaldehyde (PFA) per well for 24 h after which time the liquid contents of all wells were replaced with 50 μL PBS. Differential interference contrast (DIC) and 4′,6-diamidino-2-phenylindole (DAPI) image stacks were acquired (Fig. 2a) with 10× magnification using a Nikon Eclipse TE2000 inverted microscope (Nikon Instruments Inc.).

### Tumorsphere assay

A standardized 3D tumor spheroid growth assay was carried out as reported previously [58]. Briefly, 100 μL of the graded drug dilutions and DMSO controls were added to 96-well plates with 4000 cells in soft agar per well (n = 6 per cell line per drug concentration). After a ten-day incubation period, a resazurin assay was performed, and cells were fixed as described above. DIC and GFP z-stack images were acquired with 10× magnification using a BD Pathway 855 High-Content Imager (Becton, Dickinson and Company).

### Tumor cell injections, quantification of tumor cell load in the brain and multimodal imaging of metastasis formation

Six to eight weeks old female NOD/SCID mice were purchased from the University of Bergen animal facility. Anesthesia was induced with 3% and maintained with 1.5% isoflurane in oxygen. Mice were monitored daily and sacrificed upon signs of illness. Intracardiac injections were performed using ultrasound-guidance (5 × 10^5^ cells per 0.1 mL PBS per mouse) as reported previously [58]. H1_DL2 cells were pre-labeled with superparamagnetic iron oxide nanoparticles and MRI was carried out 24 h after injections [57]; T2*-weighted images for automated quantification of tumor cell load in the mouse brains and T2-weighted images to assess the presence of focal brain lesions. MRI equipment and sequence details have previously been described [58]. For T2*-weighted quantification we developed a cell-line specific training set for the integrated neural network analysis. Only mice with comparable tumor cell load in their brains and without ischemic lesions were included in further studies. We omitted five mice in the candidate drug study and six mice in the β-sitosterol study, based on an uneven tumor load to the brain.

**Fig. 3:**
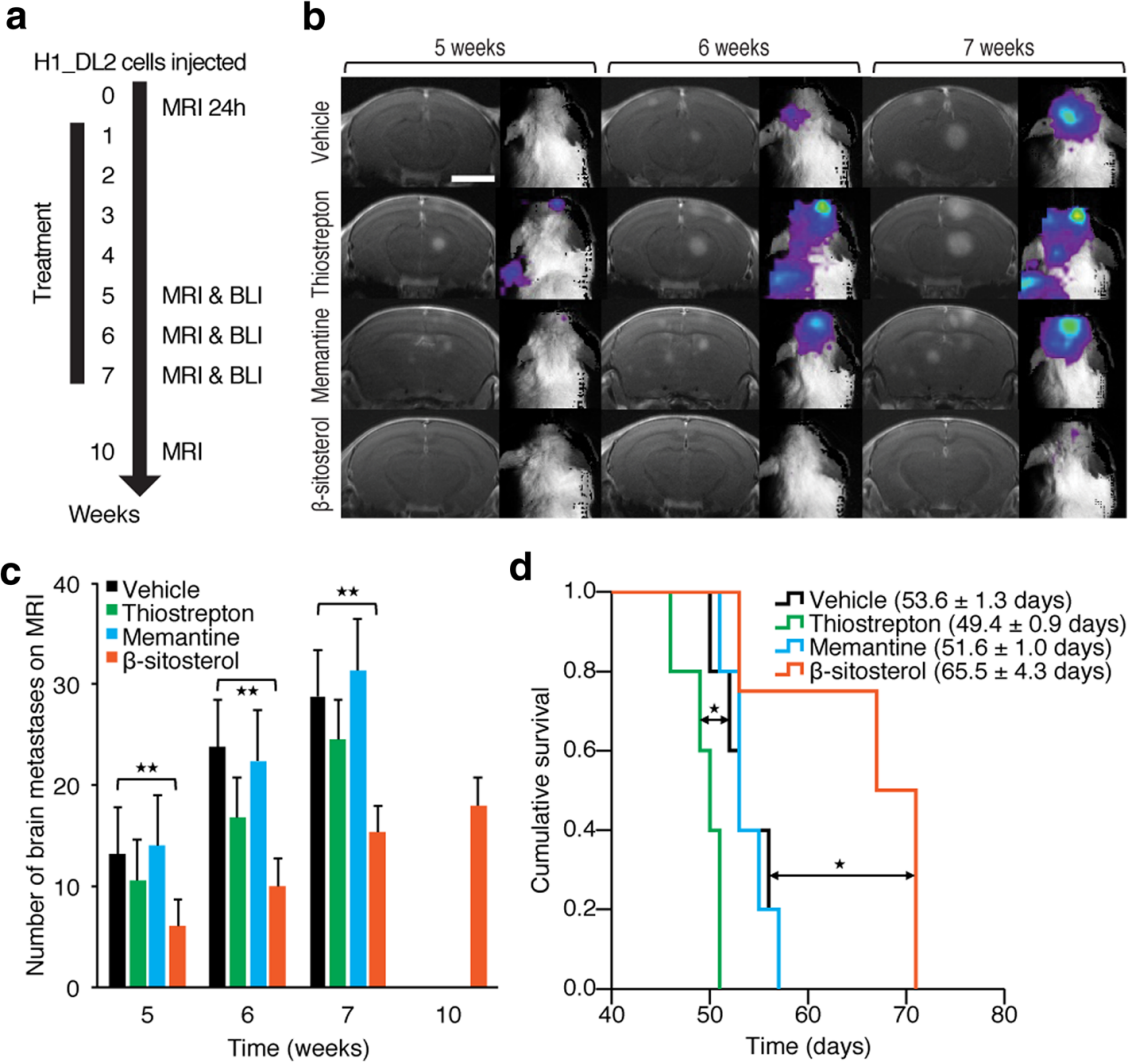
In vivo drug screening. **a** Experimental overview: Intracardiac injections of 5 × 10^5^ H1_DL2 cells in NOD/SCID mice were followed by MRI-based quantification of nanoparticle-labeled melanoma cells in the mouse brains after 24 h for group homogenization (Additional file 5: Figure S4a). Treatment started after one week, and test groups received 0.2 mL i.p. injections of 40 mg/kg thiostrepton every second day (*n* = 5), 10 mg/kg memantine daily (n = 5), 5 mg/kg β-sitosterol daily (*n* = 4) or vehicle (0.5% DMSO; n = 5). See Additional file 5: Figure S4 for more details. **b** Development of brain metastases visualized by MRI (T1-weighted images with contrast) and BLI at five, six and seven weeks. Scale bar MRIs, 0.25 cm. **c** Number of brain metastases at T1-weighted MRI with contrast (Student’s *t*-test). **d** Kaplan-Meier survival plot (Mantel-Cox log-rank test). There was no significant difference between vehicle- and memantine-treated mice. * *P* < 0.05; ** *P* < 0.01. All values are given as the mean ± s.e.m

**Fig. 4:**
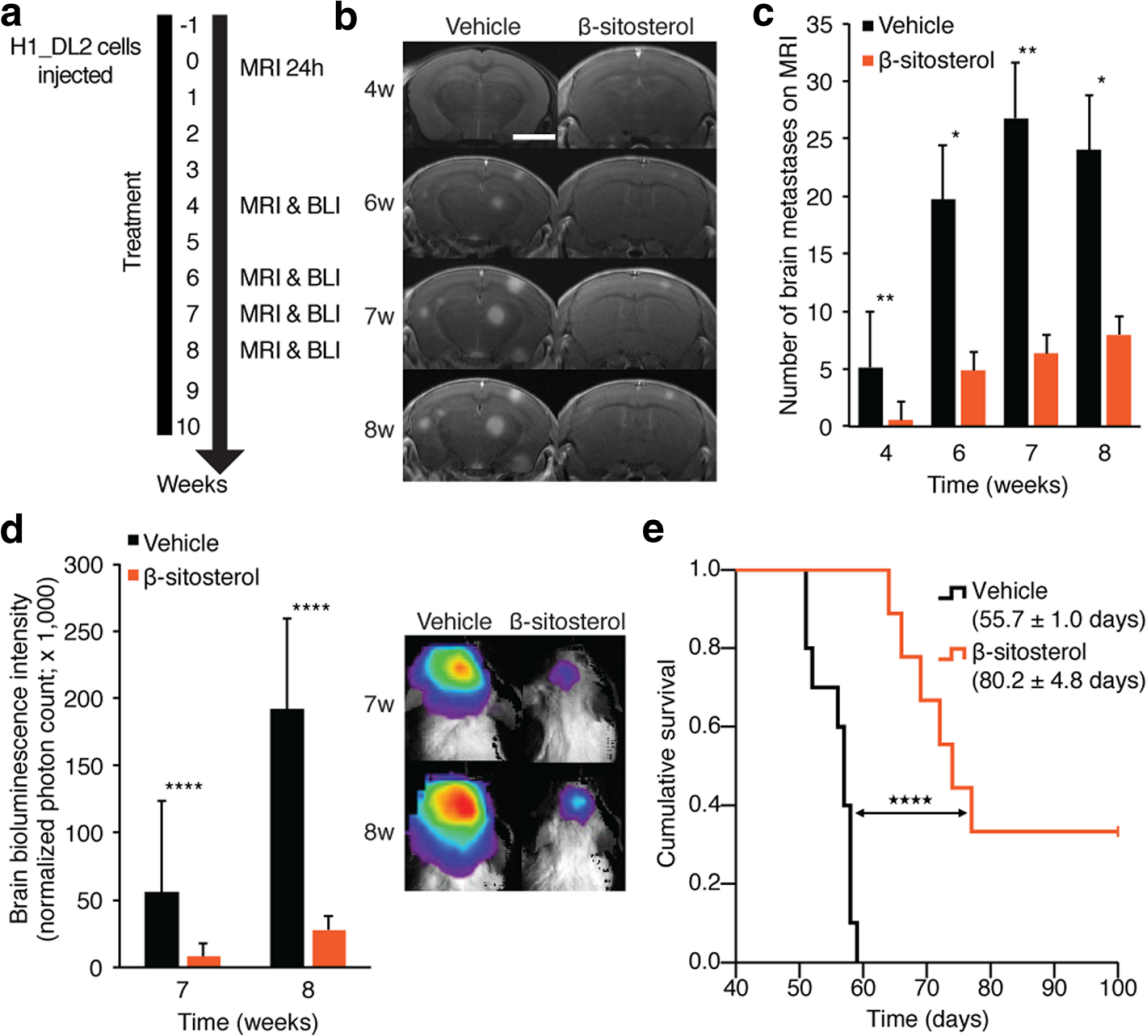
In vivo validation of β-sitosterol pre-treatment. **a** Experimental overview: Treatment started one week prior to intracardiac injections of 5 × 10^5^ H1_DL2 cells in NOD/SCID mice. Quantification of tumor cell exposure in the mouse brains was carried out 24 h after injections for group homogenization (Additional file 6: Figure S5a). Test groups received daily i.p. injections of 0.1 mL vehicle (olive oil; *n* = 10) or β-sitosterol diluted in olive oil (5 mg/kg; *n* = 9). Vehicle treatment was continued until euthanization and β-sitosterol was given for ten weeks. See Additional file 6: Figure S5 for more details on this experiment. **b** Development of brain metastases assessed by MRI at four, six, seven and eight weeks. Scale bar MRIs (T1-weighted images with contrast), = 0.25 cm. **c** Number of brain metastases assessed by T1-weighted MRI with contrast (Student’s *t*-test). The mean number of brain metastases in the vehicle group decreased slightly from seven to eight weeks as four mice with the greatest number of brain metastases were sacrificed between these observation points. **d** Brain BLI at seven and eight weeks (total photon count = dorsal + ventral region of interest (ROI); Student’s *t*-test). **e** Kaplan-Meier survival plot (Mantel-Cox log-rank test). The experiment was terminated at 100 days, and three mice in the β-sitosterol group were still alive and healthy. * *P* < 0.05; ** *P* < 0.01; **** *P* < 0.0001. All values are given as the mean ± s.e.m

We performed brain MRI to evaluate the brain metastatic burden with T2-weighted sequences and pre−/post-contrast T1-weighted sequences as previously described [58]. Images were obtained at pre-defined week numbers (Figs. 3a, b and 4a, b). The number of tumors, contrast enhancement and volume (4/3 × π × r^3^) were assessed using OsiriX 5.8.1 32-bit (Pixmeo SARL).

In parallel with the MRI, we did BLI to evaluate brain metastatic burden. Mice were imaged 10 min after i.p. injection of 150 mg/kg D-luciferin Firefly (Gold Biotechnology). To reduce distress, they were not depilated. Images were acquired using an Optix MX3 Small Animal Molecular Imager (ART Inc.) and analyzed with Optix OptiView 3.02 (ART Inc.). Total photon counts (ventral + dorsal) were registered from identically sized regions of interest (ROIs) placed over the head.

### In vivo treatment protocols

In the drug screening study on H1_DL2 brain metastases, treatment started one week post-injection and stopped at the end of week seven (Fig. 3a and Additional file 5: Figure S4). Test groups received 0.2 mL i.p. injections of 40 mg/kg thiostrepton every second day (*n* = 5), 10 mg/kg memantine daily (n = 5), 5 mg/kg β-sitosterol daily (*n* = 4), or 0.5% DMSO (n = 5) daily. Stock solutions of the respective drugs, as described above, were diluted with saline solution, pre-warmed (37 °C) and sonicated prior to i.p. injections (the partly miscible thiostrepton solution was homogenized before administration). Fresh solutions were made every second week and stored at 4 °C.

In the validation study with β-sitosterol treatment on H1_DL2 cells and PC14_PE6_Br2 brain metastases, treatment started one week prior to intracardiac injections and was continued until sacrifice for the vehicle groups (*n* = 10) and for ten weeks for the β-sitosterol groups (*n* = 9). Test groups received daily i.p. injections of 0.1 mL olive oil or 5 mg/kg β-sitosterol in 0.1 mL olive oil. β-sitosterol was mixed with olive oil (Santa Cruz Biotechnology Inc) and solubilized over 4 h with a heated magnetic stirrer (50 °C). Fresh solutions were made every second week and stored at room temperature.

In the subcutaneous tumor model, we used the Melmet 5 melanoma cell line. We injected 1 × 10^6^ cells in 0.1 mL PBS in the cervical skin fold of 32 mice. After two weeks, when the average tumor volume was approximately 30 mm^3^, the mice were randomized to daily i.p. injections of 0.1 mL vehicle (olive oil), 20 mg/kg β-sitosterol in olive oil, 25 mg/kg PLX4720 in 0.05% DMSO, or a combination of the two latter (*n* = 8 in each group). Caliper measurements were carried out every sixth day to evaluate tumor growth. Tumor volume was calculated using the formula (width^2^ × length)/2.

### Functional classification of the brain metastasis gene signature

We performed a functional classification of human biological processes and signaling pathways associated with the 108-gene signature using the Protein Analysis Through Evolutionary Relationships (PANTHER) classification system (http://www.pantherdb.org/).

### Protein interactions of β-sitosterol

We examined known and predicted protein interactions of β-sitosterol using the Search Tool for Interactions of Chemicals (STITCH 4.0; http://www.stitch.embl.de). We applied a high confidence level cut-off (0.700) and a maximal number of interactions (*n* = 50). We next investigated with which cellular processes these protein targets were associated using the Human Experimental/Functional Mapper (HEFalMp).

### Protein phosphorylation assay

For the determination of the relative levels of protein phosphorylation of 43 kinases and 2 related total proteins (HSP60 and β-Catenin), we used the Human Phospho-Kinase Array (R&D Systems, Inc.) as specified by the manufacturer. H1_DL2 cells were treated with vehicle (0.05% DMSO) or β-sitosterol (50 μM) for 24 h.

### Linkage analyses to mitochondrial metabolism

We obtained the mitochondrial metabolism genes from the Gene Ontology (GO) database using AmiGO (http://amigo.geneontology.org/amigo) and the search term «oxidative phosphorylation» (filter: *Homo sapiens*). This resulted in 121 unique gene symbols among a total of 209 gene-term associations. β-sitosterol targets were extracted from ChEMBL (Chemical Database of the European Molecular Biology Laboratory; https://www.ebi.ac.uk/chembl): 16 targets reported in humans. CHEMBL_IDs were converted to gene symbols with UnitProtKB (Universal Protein Resource Knowledgebase; http://www.uniprot.org). Network generation and visualization were implemented in Cytoscape 3.3.0 (http://www.cytoscape.org). Interactions between query and mitochondrial metabolism gene sets were extracted with ANAT (Advanced Network Analysis Tool; http://www.cs.tau.ac.il/~bnet/ANAT). β-sitosterol targets and signature genes were defined as network anchors, and the oxidative phosphorylation genes as network terminals. Anchored network analysis was applied with focus on: human interactions, protein-protein and protein-DNA interactions and default search parameters. Interaction directionality from anchors to terminals was specified.

### Extracellular flux analysis

Extracellular flux analysis with concurrent detection of oxygen (mitochondrial respiration) and pH (lactate production indicating glycolysis) was employed. The measurements were performed in 96-well H1_DL2 and HEMa cell cultures using the Seahorse XF96 Analyzer system (Seahorse Bioscience), according to manufacturer’s protocol. The assay conditions were optimized with regard to cell number and concentrations of carbonyl cyanide 3-chlorophenylhydrazone (CCCP) or carbonyl cyanide-4 (trifluoromethoxy) phenylhydrazone (FCCP) and oligomycin. Cells were seeded in quadruplicate wells (2 × 10^4^ cells per well), and exposed to 50 μM β-sitosterol for 24 h before the analysis. Control cells received 0.05% DMSO. Following this treatment, the growth medium was replaced with assay medium consisting of phenol-free DMEM supplemented with 2 mM L-glutamine and 2 mM sodium pyruvate. 10 mM glucose was added to the medium for measurement of mitochondrial respiration. The assay medium was adjusted to a pH of 7.4. The cells were incubated at 37 °C in a CO_2_-free incubator (Seahorse XF Prep station) for 1 h before they were transferred to the analyzer. To investigate mitochondrial respiratory function, assessment of the initial oxygen consumption rate (OCR), indicating the basal rate, was followed by sequential additions of several modulators: 1) the ATP synthase inhibitor oligomycin (3 μM for H1_DL2 and 2 μM for HEMa cells) was used to measure phosphorylation independent respiration (Leak); 2) the uncoupler CCCP or FCCP (1.5 or 2 μM for H1_DL2 and HEMa cells, respectively) to measure the capacity of the electron transport system (respiratory capacity); 3) the Complex I (CI) inhibitor rotenone (1 μM) to determine CI independent respiration; and 4) the CIII inhibitor antimycin A (1 μM) to assess residual background OCR not related to mitochondrial respiration; this was subtracted as background from the other measurements in the statistical analysis. To analyze the glycolytic function, the extracellular acidification rate (ECAR) was measured after sequential additions of glucose (10 mM) to determine basal glycolysis and oligomycin (3 μM) to obtain glycolytic capacity. CCCP (1.5 μM) was injected to address the possible influence of uncoupling, and 2-deoxyglucose (2-DG; 100 mM) was used to obtain the non-glycolytic background. In statistical analysis, the residual ECAR was subtracted as background from the other measurements. All experiments were performed three times.

### High-resolution respirometry

Oxygen consumption was analyzed using the Oxygraph O2K instrument and DatLab software (Oroboros Instruments). The H1_DL2 cells were harvested and washed in PBS before they were suspended in the assay medium (20 mM K-hepes, 83 mM KCl, 4 mM KH2PO4, 1 mM EGTA and 1 mM MgCl2) and transferred to the assay chamber (37 °C) at 1.25 × 10^6^ cells per 2 mL assay medium. Digitonin (8.1 μM) was added to permabilize the cell membrane. The combined CI + CII-driven respiration was obtained in the presence of malate (2 mM), pyruvate (1 mM), succinate (10 mM), and FCCP (titrated to 0.18 μM). Following injection of β-sitosterol (50 μM) or DMSO (0.05%) and assessment of the resulting OCRs, rotenone (0.5 μM) was injected to determine if the effect was linked to CI and/or CII. Antimycin A (2.3 μM) was added to provide the non-mitochondrial background activity, which was subtracted during data analysis. This experiment was performed three times.

### Measurement of reactive oxygen species and apoptosis assay

H1_DL2 cells were treated with 0.05% DMSO or 50 μM β-sitosterol for 24 h, and apoptosis was detected and quantified using the disodium salt of 3, 4, 5, 6,-tetrachloro-2′, 4′, 5′, 7′-tetraiodofluorescein (TCTF) as previously described (*n* = 3) [34]. The cellular content of reactive oxygen species (ROS) was measured using the CM-H_2_DCFDA probe according to the manufacturer’s instructions (Life Technologies). Briefly, naïve H1 cells were treated with 0.05% DMSO or 50 μM β-sitosterol for 2 h before exposure to 5 μM CM-H_2_DCFDA for 15 min (two experiments with triplicate measurements). Data were analyzed using a BD Accuri C6 flow cytometer (BD Biosciences).

### Western blot

Protein extraction and western blotting was carried out as described previously [58]. Antibodies were diluted in blocking buffer and the following antibodies were used: Caspase-3 (1:500) and PGC1α (1:500) (Cell Signaling Technology); and NDUFA8 (ab74126; 1:500), GAPDH (1:20,000) and β-actin (1:20,000) (Abcam).

### MitoTracker red fluorescence

MitoTracker Red (Invitrogen) mean fluorescence intensity was measured in H1 cells treated with DMSO (0.05%) or PLX4720 (1.5 μM) for 72 h (n = 3). Data were analyzed using a BD Accuri C6 flow cytometer (BD Biosciences).

### Colony formation assay

5 × 10^3^ H1 cells were cultured in 6-well plates and treated with DMSO (0.05%), PLX4720 (1.5 μM), β-sitosterol (50 μM), or a combination of the latter two for one (controls) or three weeks (treatment groups) (n = 3). The same protocol was used for H1_shNDUFA8 cells, but these were not treated with β-sitosterol.

### Immunohistochemistry of human brain metastases

We stained 197 formalin-fixed and paraffin-embedded human brain metastases using NDUFB8 (ab110242; Abcam). Samples were derived from melanoma (*n* = 78), non-small cell lung cancer (*n* = 52), breast cancer (*n* = 25), renal cell cancer (*n* = 9), colon cancer (n = 9), small cell lung cancer (n = 7), carcinomas not otherwise specified (n = 9) and other rare cases like ovarian and esophageal cancer (*n* = 8). All staining procedures were performed using an automated immunostainer (Leica Bond III) and analyzed by ≥ two observers using a semi-quantitative score (H-Score) ranging from 0 to 300. H-Score is defined by the staining intensity (1 = weak, 2 = moderate, 3 = strong) multiplied with the frequency of positive cells (%).

### Statistics

Statistical analyses were conducted with SPSS 21 for Mac (SPSS Inc.) and Prism 7 for Mac, Version 7.0b (La Jolla, CA, USA). An independent samples Student’s *t*-test was used to compare two normally distributed groups. The Kruskal-Wallis test was used to analyze nonparametric data and Dunn’s or Sidak’s test was used to correct for multiple testing. Levene’s test was used to assess the variance. Kaplan-Meier survival analysis and Mantel-Cox log-rank test were used to assess survival differences between groups. Values are presented as means ± standard error of the mean (s.e.m.) unless otherwise specified. A two-tailed *P* ≤ 0.05 was considered significant.

### Study approval

We obtained written informed consent before human tumor material was collected. The Regional Ethical Committee and the Norwegian Directorate of Health approved the collection and storage of human tissue. The Institutional Animal Care and Use Committee at the University of California Davis and at the University of Bergen approved the mouse experiments. At both institutions, mice were maintained in animal facilities certified by the Association for Assessment and Accreditation of Laboratory Animal Care International. The local ethical committee at the University Cancer Center Frankfurt approved the immunohistochemical analyses of human brain metastases.

## Results

### Predicting therapeutic compounds using inverse gene expression profiling

Using a highly characterized metastatic *BRAF*-mutant human melanoma xenograft model [57], we isolated and sorted tumor cells from brain, bone, adrenals and ovaries by flow cytometry (Fig. 1a, Additional file 2: Figure S2a, b). By comparing RNA-seq data from the different metastases, we identified a 108-gene brain metastasis signature using a comparative workflow of independent analyses of gene expression profiles from brain versus the other organ metastases (Fig. 1b, and Additional file 2: Figure S2c, d). The signature consisted of 54 upregulated and 54 downregulated genes (Additional file 2: Figure S2d). To identify therapeutic compounds with the potential to revert the gene signature, we queried cMap with the 108-gene brain metastasis signature. cMap revealed 1313 expression profiles, with a cMap score < 0, which represent compounds with the potential to induce the opposite transcriptional response when compared with the signature (Additional file 2: Figure S2e). For further studies, we focused on the top 10 candidate compounds (all with cMap scores < *−* 0.90) as shown in Fig. 1c.

### In vitro and in vivo drug screening reveals β-sitosterol as a potential therapeutic agent

The therapeutic efficacy of the compounds was assessed on four metastatic melanoma cell lines using monolayer viability and tumorsphere assays (Fig. 2a-c and Additional file 4: Figure S3). IC_50_ values were generally lower in tumorspheres than in monolayers. The most potent compounds for reducing viability were thiostrepton, memantine, and β-sitosterol (Fig. 2a, b). The IC_50_ values for normal human fibroblasts and astrocytes were 128.3 μM and 1200.5 μM respectively, whereas normal human melanocytes were not sensitive to β-sitosterol within the dose range studied (Additional file 4: Figure S3).

We next assessed the therapeutic efficacy of thiostrepton, memantine, and β-sitosterol using a highly standardized human-to-mouse brain metastasis model [57] (Fig. 3a). Quantitative analysis of baseline H1_DL2 melanoma cells (i.e. accumulation of tumor cells within the brain 24 h after injection) showed no significant differences between the treatment and vehicle (control) groups (Additional file 5: Figure S4a). Drug treatment was commenced one week after tumor cell injections. High-resolution magnetic resonance imaging (MRI) over the next 5–10 weeks revealed a significant reduction in the number and volume of brain metastases in the β-sitosterol-treated mice when compared with vehicle-treated mice (Fig. 3b, c and Additional file 5: Figure S4b). There were no differences in brain metastasis frequency or size between the vehicle and the thiostrepton or memantine groups (Student’s *t*-test, *P* ≥ 0.05 for all comparisons). BLI intensity was significantly lower for the β-sitosterol group when compared to the vehicle group at all time points, with the exception of brain BLI intensity at five weeks (Student’s *t*-test, *P* ≥ 0.05; Additional file 5: Figure S4c). Importantly, β-sitosterol-treated mice survived significantly longer than vehicle-treated mice, whereas thiostrepton-treated mice had a significantly shorter lifespan. Also, memantine treatment did not affect survival (Fig. 3d).

### β-Sitosterol pre-treatment inhibits the formation of brain metastasis and increases animal survival

To further validate the β-sitosterol treatment effect, we performed a new and more extensive in vivo study where β-sitosterol treatment started 1 week before tumor cell injection (Fig. 4a). MRI-based quantification of baseline H1_DL2 melanoma cells in mouse brains again showed an equal tumor cell exposure in the β-sitosterol and vehicle groups (Additional file 6: Figure S5a). Vehicle-treated mice progressively lost weight from four weeks and onwards, whereas β-sitosterol-treated mice maintained a stable body weight (Additional file 6: Figure S5b). There were significantly fewer and smaller brain metastases in the β-sitosterol group than in the vehicle group (Fig. 4b, c and Additional file 6: Figure S5c). Brain BLI intensity was significantly lower in the β-sitosterol treatment group compared to the vehicle group at comparable time points (Fig. 4d). β-sitosterol treatment provided a significant survival benefit when compared with vehicle treatment (Fig. 4e). When the study was terminated at 100 days, three out of nine mice treated with β-sitosterol were healthy and tumor-free as evaluated by brain MRI and histology. To confirm that the effects observed were not associated with the model used, we performed the same study using another highly aggressive brain tropic cell line (PC14_PE6_Br2). Also here, a significant reduction in brain metastatic burden was seen leading to a significant improved survival (Additional file 7: Figure S6).

### β-Sitosterol targets mitochondrial respiration through complex I inhibition

To obtain mechanistic insight into the therapeutic action of β-sitosterol, we first examined the biological processes and signaling pathways associated with our brain metastasis signature. Functional classification of the signature genes showed an association with human metabolic processes and a number of cancer-related signaling pathways (Additional file 8: Figure S7a, b). We next examined known and predicted protein interactions of β-sitosterol and found direct interactions with 12 proteins within two distinct clusters related to apoptosis and cholesterol homeostasis (Additional file 8: Figure S7c). These 12 proteins acted in conjunction and were significantly associated with several biological processes, particularly metabolism and cell division (Additional file 8: Figure S7d). In order to explore whether specific signal transduction pathways were affected through the treatment with β-sitosterol, we performed a phosphorylation screen in vitro*,* and found reduced phosphorylation levels of a large number of oncogenic kinases following β-sitosterol treatment (Additional file 9: Figure S8). These data connected major regulators of cell homeostasis to the therapeutic potential of β-sitosterol and suggested that β-sitosterol may interfere with basic cellular functions such as energy metabolism and apoptosis.

**Fig. 5:**
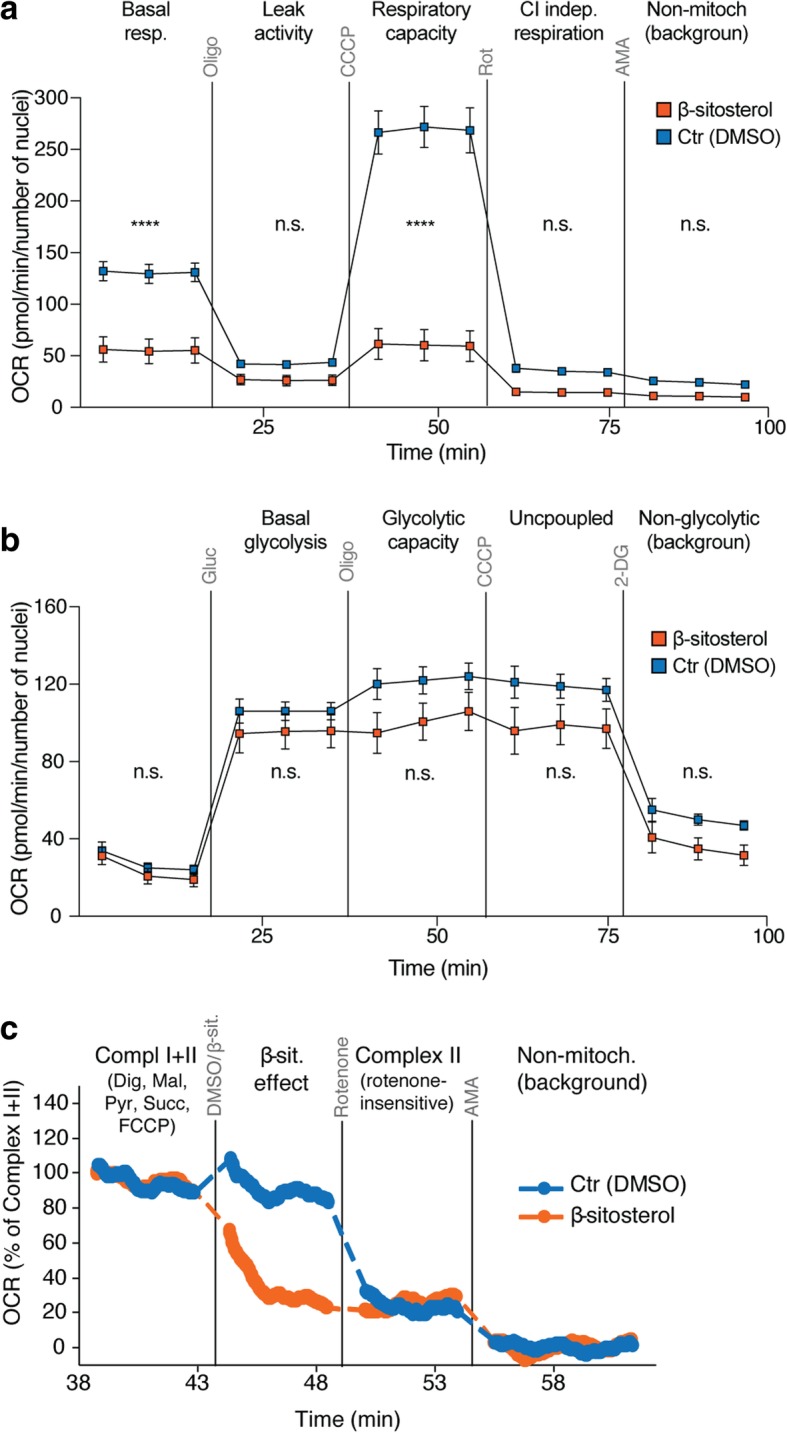
β-sitosterol reduces mitochondrial respiration through complex I inhibition. **a-b** Oxygen consumption rate (OCR) and extracellular acidification rate (ECAR) were measured to assess rates of mitochondrial respiration and glycolysis, respectively, in H1_DL2 cells treated with 50 μM β-sitosterol or 0.05% DMSO for 24 h (both: n = 4). **a** Basal respiration was determined, followed by sequential additions of oligomycin (3 μM) to assess respiration due to proton leak, carbonyl cyanide 3-chlorophenylhydrazone (CCCP; 1.5 μM) to measure respiratory capacity, rotenone (1 μM) to assess Complex I (CI) independent respiration and antimycin A (AMA; 1 μM) to determine background OCR. **b** Glucose (10 mM) was provided to determine basal glycolysis, followed by sequential additions of oligomycin (3 mM) to obtain glycolytic capacity, CCCP (1.5 μM) to evaluate the influence of uncoupling and 2-deoxyglucose (2-DG; 100 mM) to measure the non-glycolytic background. **c** High-resolution respirometry in H1_DL2 cells to detect direct effects of β-sitosterol. First, the maximal CI + CII driven respiratory capacity was measured in the presence of digitonin (8.1 μM), malate (2 mM), pyruvate (1 mM), succinate (10 mM) and carbonylcyanide-4-(trifluoromethoxy)-phenylhydraqone (FCCP, 0.18 μM). The respiratory rate was then measured after adding β-sitosterol (50 μM) or DMSO (0.05%), followed by rotenone (0.5 μM) to inhibit CI, and AMA (2.3 μM) to determine residual oxygen consumption. The experiment was repeated 3 times. **a**-**c** Student’s *t*-test: n.s. = not significant, *P* ≥ 0.05, **** *P* < 0.0001. Values are given as the mean ± s.d

Previous studies have shown that metastatic cells adapt their energy production in order to thrive in the brain microenvironment by increasing their mitochondrial respiration. This process has been shown to be a key mediator of resistance to BRAFi [15, 16, 24, 63]. We therefore investigated, by bioinformatics analyses, protein-protein and protein-DNA interactions between the 121 Gene Ontology-annotated genes implicated in oxidative phosphorylation and (a) our brain metastasis signature or (b) known β-sitosterol targets. These analyses revealed large interaction networks with centrally located signature genes (Additional file 10: Figure S9) and β-sitosterol targets (Additional file 11: Figure S10). These data indicate that the therapeutic effect of β-sitosterol is linked to mitochondrial interference. Thus, we measured mitochondrial respiration and glycolysis by extracellular flux analysis in H1_DL2 melanoma cells following β-sitosterol treatment. As shown in Fig. 5a, β-sitosterol strongly reduces basal mitochondrial respiration and respiratory capacity. The extracellular flux analysis further shows that inhibition of ATP synthase (with oligomycin) is similar in vehicle- and β-sitosterol-treated cells (Fig. 5a), indicating that β-sitosterol does not disrupt the integrity of the mitochondrial inner membrane. Inhibition of respiratory CI revealed that most of the respiratory activity is linked to this complex (Fig. 5a) and importantly, suggested that CI was a likely β-sitosterol target. Basal glycolysis and glycolytic capacity were, however, unaffected by β-sitosterol (Fig. 5b). Interestingly, melanoma cells showed minimal glycolytic reserve (glycolytic capacity minus basal glycolysis) if mitochondrial ATP production should cease (Fig. 5b). Thus, the cells could be particularly sensitive to inhibitors of mitochondrial respiration such as β-sitosterol. For comparison, we also measured the respiratory capacity of normal melanocytes following β-sitosterol treatment. Compared to the tumor cells, no changes in respiratory capacity was observed (Additional file 12: Figure S11).

To determine if the inhibitory effect of β-sitosterol on mitochondrial respiration was directly linked to the activity of CI or CII, we analyzed oxygen consumption rates in permeabilized cells by high-resolution respirometry. The CI + CII-driven respiratory activity was inhibited immediately following β-sitosterol exposure (Fig. 5c). Addition of the CI inhibitor, rotenone, did not provide further inhibition, and the remaining CII-driven rate was similar in the presence of β-sitosterol or rotenone (Fig. 5c). In summary, β-sitosterol inhibits mitochondrial respiration in tumor cells by acting as a CI inhibitor. Such an inhibition was not seen in normal melanocytes.

### β-Sitosterol increases oxidative stress and induces apoptosis

**Fig. 6:**
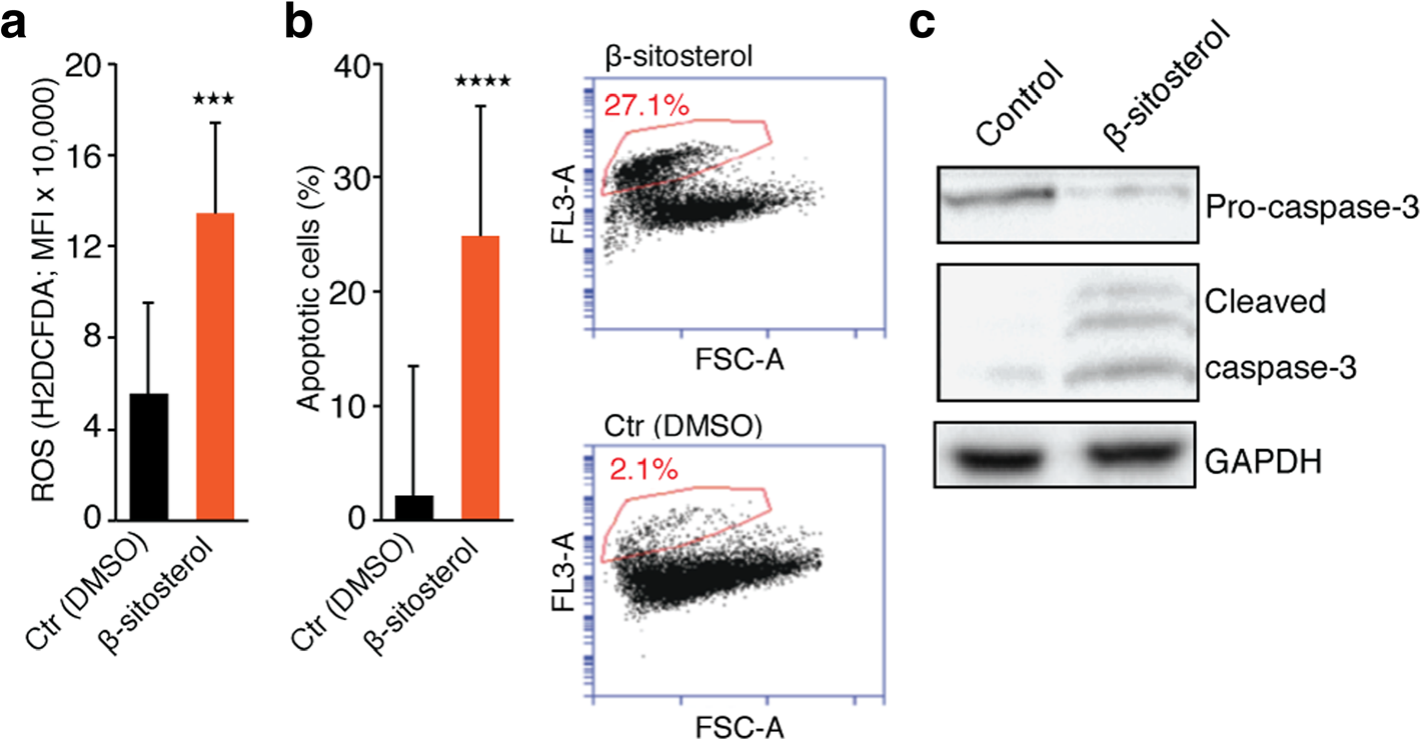
β-sitosterol increases ROS production and apoptosis. **a** ROS content (CM-H_2_DCFDA probe; mean fluorescence intensity (MFI); *n* = 2 with triplicates). **b** Flow cytometric apoptosis assay (n = 3) showing a strong induction of apoptosis following ß-sitosterol treatment. **c** Western blot of pro-caspase-3, cleaved caspase-3 and GAPDH in H1_DL2 cells exposed to DMSO (0.05%) or β-sitosterol (50 μM) for 2, 24 or 24 h, respectively. Student’s *t*-test: *** *P* < 0.001, **** *P* < 0.0001. Values are given as the mean ± s.e.m

Beyond their function in ATP synthesis, mitochondria are major producers of ROS. CI respiratory capacity is of particular importance in this respect, −and inhibition of its activity often results in increased ROS production [36]. Consistent with previous studies [4, 50], we observed a significant increase in cellular ROS content following β-sitosterol treatment (Fig. 6a). Interestingly, recent observations have shown that oxidative stress inhibits metastatic melanoma cells in the blood and visceral organs in vivo [42]. Since increased ROS levels have been linked to apoptosis induction, we next performed apoptosis analyses. As shown in Fig. 6b, a significant induction of apoptosis was observed following β-sitosterol treatment. This is in line with previous studies showing that β-sitosterol can induce both mitochondrial- and death receptor-mediated apoptosis in cancer cells [4, 8, 23, 50, 64, 65, 70]. Additionally, immunoblots for apoptotic markers (Fig. 6c) confirmed our protein interaction analysis (Additional file 8: Figure S7c). In summary, respiratory capacity is inhibited following β-sitosterol treatment which leads to an induction of apoptosis. In this context, it should also be emphasized that apoptosis is a hallmark of MAPK-targeted therapies as well as mitochondrial inhibitors, including known inhibitors of CI-mediated respiration [24, 36, 68].

### β-Sitosterol abrogates potential resistance to BRAF inhibition

**Fig. 7:**
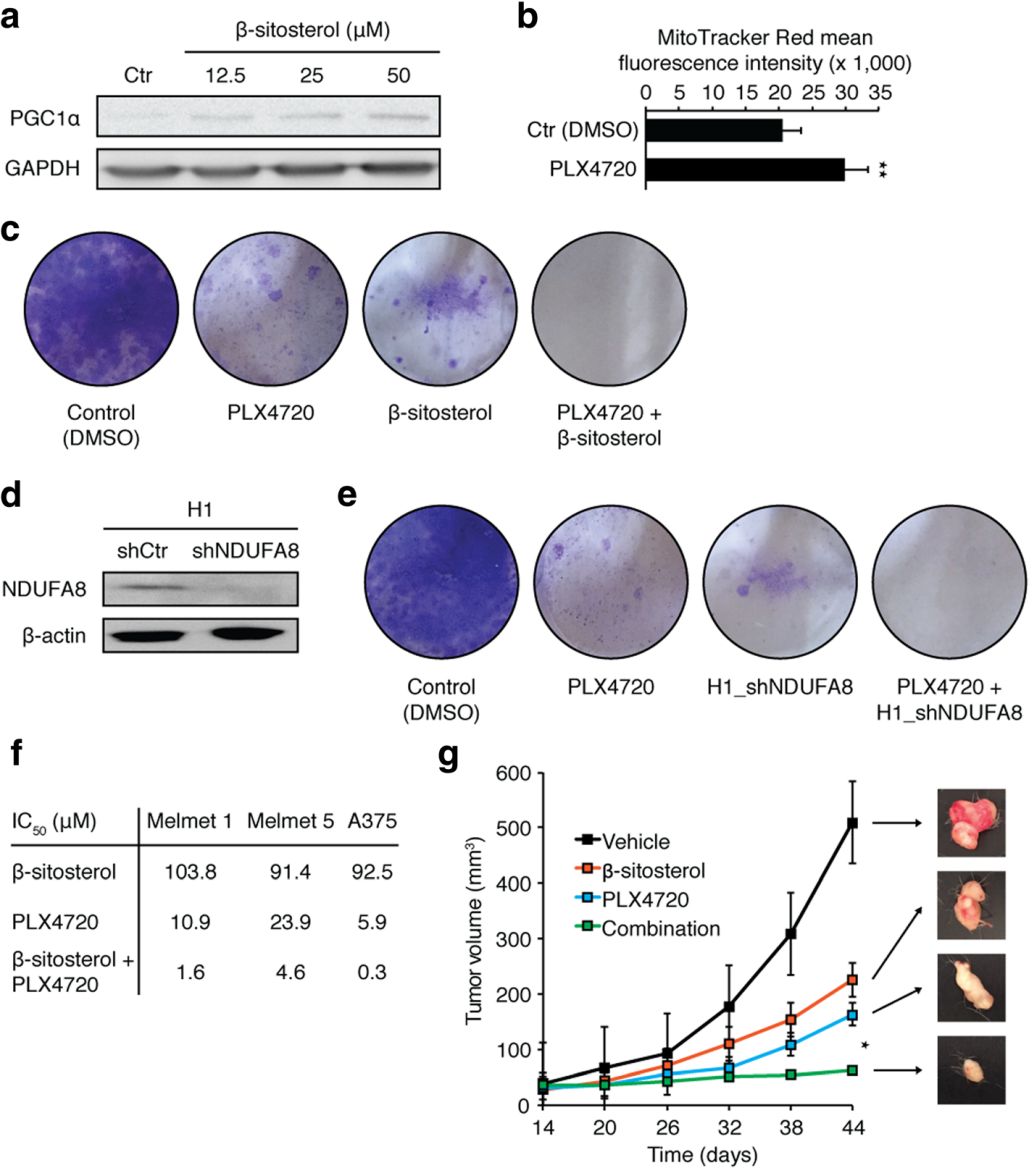
Mitochondrial complex I inhibition prevents BRAFi resistance. **a**. Western blot of PGC1α and GAPDH in H1 cells treated with DMSO (0.05%) or β-sitosterol (12.5, 25 or 50 μM) for 24 h (n = 3). **b**. MitoTracker Red mean fluorescence intensity in H1 cells treated with DMSO (0.05%) or vemurafenib (1.5 μM) for 72 h (n = 3). **c** Colony formation assay (crystal violet staining) of H1 cells treated with DMSO (0.05%) for 1 week, or vemurafenib (1.5 μM), β-sitosterol (50 μM), or vemurafenib + β-sitosterol for 3 weeks (n = 3). **d** Western blot of NDUFA8 and β-actin in H1_shCtr and H1_shNDUFA8 cells (NDUFA8 is required for assembly of a functional complex I). **e** Colony formation assay of H1 cells treated with DMSO (0.05%) for one week or vemurafenib (1.5 μM) for three weeks, and H1_shNDUFA8 cells alone or treated with vemurafenib (1.5 μM) for three weeks (n = 3). **f** Half maximal inhibitory concentration (IC_50_) values for β-sitosterol, PLX4720 and the combination thereof in Melmet 1, Melmet 5 and A375 cell lines (n = 3). **g** Subcutaneous tumor volume (width^2^ × length)/2) in mice injected with 1 × 10^6^ Melmet 5 cells. Mice were from two weeks onwards given daily i.p. injections of 0.1 mL vehicle (olive oil), 20 mg/kg β-sitosterol in olive oil, 25 mg/kg PLX4720 in 0.05% DMSO, or a combination of the two latter (*n* = 8 in each group). Representative images of tumors at 44 days are shown in the right panel. Student’s *t*-test: * *P* < 0.05. Values are given as the mean ± s.e.m

Consistent with our previous observations that β-sitosterol inhibits mitochondrial respiration (Fig. 5a) and increases oxidative stress (Fig. 6a), we found a compensatory increase in PGC1α expression with increasing concentrations of β-sitosterol (Fig. 7a). The MITF-PGC1α axis is a master regulator of mitochondrial function in melanomas [24, 63]. PGC1α promotes mitochondrial respiration and protects against oxidative stress. A subset of melanomas overexpresses PGC1α, and treatment of *BRAF*-mutant melanomas with the BRAFi PLX4720 (a vemurafenib analogue) has been shown to upregulate PGC1α [24, 63]. In agreement with these data, we found that PLX4720 induced MitoTracker Red fluorescence, a measure of mitochondrial activity and mass (Fig. 7b). Importantly, these intrinsic and acquired survival advantages render melanoma cells resistant to BRAFi, and combined approaches that exploit the resultant dependence on mitochondrial respiration are highly sought [24, 45, 49, 63, 69].

We therefore investigated the possible therapeutic benefits of combining BRAFi and mitochondrial CI inhibitors in colony formation assays. Both PLX4720 and β-sitosterol were effective as monotherapies, but regrowth of resistant clones appeared after long-term incubation (Fig. 7c). Combination treatment with PLX4720 and β-sitosterol, however, completely inhibited cell growth (Fig. 7c). To further substantiate the therapeutic potential of targeting mitochondrial CI (Fig. 5a, c), we constructed a stable NDUFA8 knockdown in the H1 melanoma cell line (H1_shNDUFA8) (Fig. 7d). NDUFA8 is an accessory subunit of mitochondrial CI, in which knockdown results in failure of a functional complex and severe respiratory deficiency [55]. Long-term colony formation assays with PLX4720 alone or H1_shNDUFA8 alone displayed attenuated growth, whereas the combination totally eliminated cell growth (Fig. 7e). Next, we examined whether or not the combined effect was cell line specific. We performed monolayer viability assays in three other melanoma cell lines. Combination treatment with β-sitosterol and PLX4720 substantially reduced the IC_50_ values for all cell lines (Fig. 7f). As BRAFis in general have limited penetrability through an intact BBB [37] and the BBB is commonly not degraded in animal models until late in metastatic development [60], we carried out a proof of concept study in a subcutaneous Melmet 5 melanoma model. Both PLX4720 and β-sitosterol were effective as monotherapies, but combination treatment significantly reduced tumor growth (Fig. 7g).

To evaluate CI activity and the potential clinical relevance of our findings, we performed immunohistochemical staining of NDUFB8 (accessory subunit of CI) in 197 human brain metastases from various cancers. Intriguingly, we found the highest expression levels for melanoma brain metastases (Additional file 13: Figure S12a, b). There was no difference in NDUFB8 expression between BRAF mutated and BRAF wild type melanoma brain metastases (Additional file 13: Figure S12c). Taken together, our data suggest that β-sitosterol prevents a key resistance mechanism to BRAFi therapy and may therefore be a beneficial therapeutic adjuvant in the treatment of melanoma brain metastasis.

## Discussion

In this study, we used computational predictions based on gene expression analyses to identify potential drugs against *BRAF*-mutant melanoma brain metastases. The cholesterol analogue β-sitosterol was well tolerated, effectively reduced the number and volume of brain metastases, and improved survival in reproducible and predictive human-to-mouse brain metastasis models. As shown in Additional file 9: Figure S8, β-sitosterol may assert its function on many biological processes. From a therapeutic viewpoint, targeted therapies may have a limited effect, based on their complexity (heterogeneity and numerous cell signaling events occurring in melanomas). In the current work, we have shown that β-sitosterol has a particular effect on BRAF-driven tumors, but this does not exclude an action of β-sitosterol on other biological processes in tumor cells. For instance, the PC14_PE6_Br2 lung adenocarcinoma cell line, which is BRAF wild type, was also sensitive to β-sitosterol treatment, but to a lesser extent. We found in particular that the compound inhibited mitochondrial respiration through targeting of mitochondrial CI, which is a major facilitator of intrinsic and acquired resistance to MAPK-targeted therapies [24, 63]. Importantly, we also found that the combination of β-sitosterol and a BRAFi exhibited a strong therapeutic effect, compared to monotherapies.

Regarding a potential translational value of the presented findings, the preventive effect of β-sitosterol on the establishment of brain metastasis should be highlighted (Fig. 4). Since patients with melanomas and lung cancer show a significant propensity to develop brain metastasis during disease progression, β-sitosterol may, in a preventive setting, inhibit brain metastasis from the primary tumor.

To our knowledge, this is the first example of successful preclinical repurposing of a drug to treat melanoma-associated brain metastases. Repurposing of approved, non-anticancer drugs is an attractive drug discovery strategy in cancer with substantial advantages of cheaper, faster, and safer preclinical and clinical validation [11]. It should also be emphasized that high doses (3–4 g) of β-sitosterol can be given daily to patients without side effects [30].

The phytosterol β-sitosterol is known as a competitive inhibitor of intestinal cholesterol uptake [30]. Phytosterols are generally classified as safe by the United States Food and Drug Administration, and the European Foods Safety Authority has concluded that a daily phytosterol and/or phytostanol intake of 1.5–2.4 g can reduce blood cholesterol by 7–10.5% and sustain this effect for up to 85 weeks [13]. Notably, we used a daily dose of 5 mg/kg in our experimental studies, which translates into 375 mg for a person weighing 75 kg. Randomized controlled trials (RCTs) in humans have found beneficial effects of β-sitosterol (and its ester) on hypercholesterolemia, but also on benign prostatic hyperplasia, androgenic alopecia, and as an adjuvant in the treatment of tuberculosis, and anogenital warts [9, 20, 35, 43, 53]. No clinical trials have examined the effects of β-sitosterol on cancer. However, epidemiological studies have suggested that increased consumption of phytosterols can reduce the risk of different cancers [19, 33, 38, 61]. Data from cancer cell lines have shown that β-sitosterol can reduce cell proliferation and induce apoptosis in addition to inhibiting adhesion, invasion, and migration [2, 4–6, 8, 50, 64, 65, 70]. β-sitosterol has also been shown to inhibit the growth of tumor xenografts, reduce progression of carcinogen-induced tumors, and prevent metastatic lung and lymph node colonization [5, 8, 28, 29, 54]. The anticancer effects of β-sitosterol remain somewhat elusive, but a number of underlying mechanisms have been proposed [23]. As a potential therapeutic agent against brain metastases, it is particularly interesting that β-sitosterol has been shown to cross the BBB and accumulate in the brain [25, 62]. With respect to our findings, it has previously been shown that β-sitosterol can incorporate into the inner mitochondrial membrane where it increases membrane fluidity [47]. It is also known that cancer cells may have an altered cholesterol loading, that may impair mitochondrial function leading to apoptosis protection [44]. Further studies on how β-sitosterol causes metabolic alterations in cancer cells are therefore warranted.

We found that, β-sitosterol displays broad-spectrum effects at both the genomic, proteomic, and metabolomic levels. Although extensive suppression of the MAPK pathway appears to be particularly important for metastatic melanoma, we also delineated a highly relevant mechanism of β-sitosterol, namely the reduction of mitochondrial respiration through CI inhibition with concurrent induction of ROS leading to apoptosis. This mitochondrial inhibition may have important ramifications for patients with metastatic melanoma, as increased mitochondrial oxidative capacity has been shown to mediate resistance to MAPK-targeted therapeutics and provide protection against oxidative damage and apoptosis [24, 63, 68]. Previous studies have indicated a therapeutic potential of various mitochondrial inhibitors and CI inhibitors in melanoma [21, 24, 36, 45, 49, 63, 68, 69]. In *BRAF*-mutant melanoma brain metastasis, we show that β-sitosterol efficiently prevented resistance to BRAFi therapy in vivo. Furthermore, large-scale analyses of human brain metastases indicated a significant role of mitochondrial CI, which warrants further investigation*.* Intriguingly, emerging evidence suggests that mitochondrial respiration may be a particularly important survival mechanism and growth facilitator for metastatic cells in the brain microenvironment [7, 15, 16, 26].

## Conclusions

In conclusion, we here leveraged robust in vivo model systems of brain metastasis to demonstrate the effects of β-sitosterol on *BRAF*-mutant melanoma [57]. Our study also indicates a therapeutic potential beyond brain metastasis that warrants further exploration in site-specific model systems. Importantly, to accomplish translational advances in brain metastasis research, there is a strong need for more preventive trials in selected high-risk patients or in patients with limited brain involvement [12]. Many metabolic modulators, including natural compounds and drugs used for conditions other than cancer, have favorable cost and toxicity profiles and might offer additional therapeutic benefit in metastatic melanoma. β-sitosterol can readily penetrate the BBB and has been studied in several randomized clinical trials of non-cancerous diseases [9, 20, 25, 35, 43, 53, 62]. Thus, our findings strongly encourage further assessment of β-sitosterol as an adjuvant to established MAPK-targeted therapies for patients with melanoma brain metastases or patients at risk of developing such metastases.

## Additional files

Additional file 1: **Figure S1.** The diagram illustrates the step-by-step workflow and analysis strategy used in the current study. (TIF 1390 kb)

Additional file 2: **Figure S2.** Generation of organ samples for RNA sequencing, brain metastasis gene signature and Connectivity Map analysis. a BLI five weeks after intracardiac injection of 5 × 10^5^ H1_DL2 cells in NOD.CB17-*Prkdc*^*scid*^/NcrCrl mice (*n* = 7). M1-M7, indicate mouse 1 to 7. b Fluorescence-activated cell sorting (FACS) of GFP-positive tumor cells from the metastatic lesions. The sorted cells were analyzed by RNA-seq (n = 7). c Heatmap of the 108-gene brain metastasis signature with organ samples in rows and genes in columns. d The brain metastasis gene signature with the left two panels showing 54 upregulated genes in brain metastases and the right two panels showing 54 downregulated genes. e Query results from the Connectivity Map (cMap) database using the 108-gene signature. A score of < 0 to − 1 means net reversal of the signature (negative correlation) and a score of > 0 to + 1 means net induction of the signature (positive correlation). (TIF 2033 kb)

Additional file 3: **Table S1.** Tissue Digestion Protocols. Preparation of Liberase TM Research Grade (Roche Applied Science) working solution and organ-tailored protocols for tissue digestion (brain, adrenals, ovaries and bone). (DOCX 18 kb)

Additional file 4: **Figure S3.** Cell viability in vitro by increasing β-sitosterol concentrations. Human melanoma cell lines: a Melmet 1, b Melmet 5, and c A375 (*n* = 18 per cell line per drug per drug concentration). Normal cells: d SV-80 human lung fibroblasts (n = 18 per drug concentration), e hTERT immortalized melanocytes and f Human astrocytes. All values are given as the mean ± s.e.m. (TIF 943 kb)

Additional file 5: **Figure S4.** In vivo efficacy of candidate compounds on H1_DL2 brain metastases. a Left panel: Tumor cell load into the brain was quantified by MRI-based automated quantification of nanoparticle-labeled cells in NOD/SCID mouse brains 24 h after intracardiac injection of 5 × 10^5^ H1_DL2 cells. Right panel: Images show typical MRI T2*-weighted images of mouse brains (scale bar, 0.25 cm) with an overlay of detected signals (blue). Study groups received 0.2 mL i.p. injections of 40 mg/kg thiostrepton every second day (*n* = 5), 10 mg/kg memantine daily (n = 5), 5 mg/kg β-sitosterol daily (*n* = 4) or vehicle (0.5% DMSO; n = 5) from one week post-injection. See Fig. 3 for more details on this experiment. b Volume of brain metastases determined by MRI (T1-weighted with contrast). Total volume of brain metastases per animal was compared at group level. c Brain BLI (total photon count = dorsal + ventral region of interest (ROI). a-c Student’s *t*-test: n.s. *P* ≥ 0.05, * *P* < 0.05, ** *P* < 0.01, *** *P* < 0.001. All values are given as the mean ± s.e.m. (TIF 3422 kb)

Additional file 6: **Figure S5.** In vivo validation of β-sitosterol on H1_DL2 brain metastasis. a Initial tumor cell load into the brain assessed by MRI-based automated quantification of nanoparticle-labeled cells 24 h after intracardiac injection of 5 × 10^5^ H1_DL2 cells. Images show typical MRI T2*-weighted images of mouse brains (scale bar, 0.25 cm) with an overlay of detected signals (blue). The study groups received daily i.p. injections of 0.1 mL vehicle (olive oil; *n* = 10) or β-sitosterol 5 mg/kg diluted in olive oil (*n* = 9) starting one week before tumor cell injections. See Fig. 4 for more details on this experiment. b Body weight from week zero to eight. At 100 days, the three remaining β-sitosterol mice had an average body weight of 26.7 ± 0.6 g. c Volume of brain metastases assessed by MRI (T1-weighted with contrast). Total volumes of brain metastases per animal were compared at group level. a-c Student’s *t*-test: n.s. *P* ≥ 0.05, * *P* < 0.05, ** *P* < 0.01, *** *P* < 0.001, **** *P* < 0.0001. All values are given as the mean ± s.e.m. (TIF 726 kb)

Additional file 7: **Figure S6.** β-sitosterol treatment effect is not model specific. a Experiment overview. Mice were injected intracardially with 5 × 10^5^ PC14_PE6_Br2 cells. Test groups received daily i.p. injections of 0.1 mL vehicle (olive oil; n = 4) or β-sitosterol 5 mg/kg (*n* = 6). Treatment started one week prior to tumor cell injections and was continued until euthanization. b Body weight from week zero to four for vehicle and β-sitosterol-treated mice. c Number and d volume of brain metastases at MRI at three weeks (T1-weighted with contrast). Total volume of brain metastases per animal compared at group level. e Brain BLI at three weeks (total photon count = dorsal + ventral region of interest). b-e Student’s *t*-test: n.s. *P* ≥ 0.05, * *P* < 0.05, ** *P* < 0.01, *** *P* < 0.001. f Kaplan-Meier survival plot (Mantel-Cox log-rank test: *** *P* < 0.001). All values are given as the mean ± s.e.m. (TIFF 2165 kb)

Additional file 8: **Figure S7.** a Functional classification of the brain metastasis gene signature. Human biological processes (metabolic processes are highlighted in red), and b signaling pathways associated with the 108-gene brain metastasis signature using the Protein Analysis Through Evolutionary Relationships (PANTHER) classification system. c Known and predicted protein interactions of β-sitosterol from the Search Tool for Interactions of Chemicals (STITCH 4.0). Green lines represent direct interactions with β-sitosterol whereas blue lines represent protein-protein interactions. Direct activation (green arrows) or inhibition (red lines) is indicated. d Significant statistical associations of β-sitosterol targets with cellular processes predicted with the Human Experimental/Functional Mapper (HEFalMp). *P*-values represent approximate multiple hypothesis corrected values. (TIF 1333 kb)

Additional file 9: **Figure S8.** Protein phosphorylation screening following β-sitosterol treatment. Parallel determination of protein phosphorylation levels (43 oncogenic kinases and two total proteins) in H1_DL2 cells after 24 h treatment with vehicle (0.05% DMSO) or β-sitosterol (50 μM). (TIF 418 kb)

Additional file 10: **Figure S9.** Interaction network of signature genes and oxidative phosphorylation. The 121 Gene Ontology (GO)-annotated genes implicated in oxidative phosphorylation (red nodes) have 163 experimentally validated protein-protein and protein-DNA interactions involving 143 genes/proteins, including eight signature genes (green nodes). Purple nodes: Other genes that mediate interactions between signature and oxidative phosphorylation genes. Interaction directionality is represented with arrows. (TIF 1057 kb)

Additional file 11: **Figure S10.** Interaction network of β-sitosterol targets and oxidative phosphorylation. The 121 Gene Ontology (GO)-annotated genes implicated in oxidative phosphorylation (red nodes) have 155 experimentally validated protein-protein and protein-DNA interactions involving 141 genes/proteins, including four β-sitosterol targets (green nodes). Purple nodes: Other genes that mediate interactions between β-sitosterol targets and oxidative phosphorylation genes. Interaction directionality is represented with arrows. (TIF 1056 kb)

Additional file 12: **Figure S11.** The respiratory capacity (OCR) in normal melanocytes following β-sitosterol treatment as measured by the Seahorse system. Compared to the tumor cells (Fig. 5a) no changes in respiratory capacity was observed. (TIF 263 kb)

Additional file 13: **Figure S12.** Mitochondrial complex I activity in human brain metastases. a Expression levels of complex I subunit NDUFB8 in 197 human brain metastases from different cancers: Carcinoma not otherwise specified (NOS; n = 9), colon cancer (n = 9), breast cancer (*n* = 25), renal cell cancer (RCC; n = 9), non-small cell lung cancer (NSCLC; *n* = 52), small cell lung cancer (SCLC; *n* = 7), melanoma (*n* = 78) and others (*n* = 8). H-Score = [staining intensity (1 = weak, 2 = moderate, 3 = strong)] x [frequency of positive cells (%)]. Kruskal-Wallis test with Dunn’s multiple comparisons test: * *P* < 0.05, ** *P* < 0.01, **** *P* < 0.0001 and for comparisons not indicated *P* ≥ 0.05. b Immunohistochemistry of NDUFB8 in a human melanoma brain metastasis. c Expression levels of complex I subunit NDUFB8 in BRAF mutated melanoma (BRAFV600E; *n* = 24) and BRAF wild type melanoma (no BRAFV600E; *n* = 23). Kruskal-Wallis test with Dunn’s multiple comparisons test: n.s.: not significant. (TIFF 12141 kb)
