# Supplementary figures and images for: Inhibition of mitochondrial respiration prevents BRAF-mutant melanoma brain metastasis
Source: Acta Neuropathol Commun. 2019 Apr 10;7:55. doi: 10.1186/s40478-019-0712-8 (PMC6456988; doi:10.1186/s40478-019-0712-8)

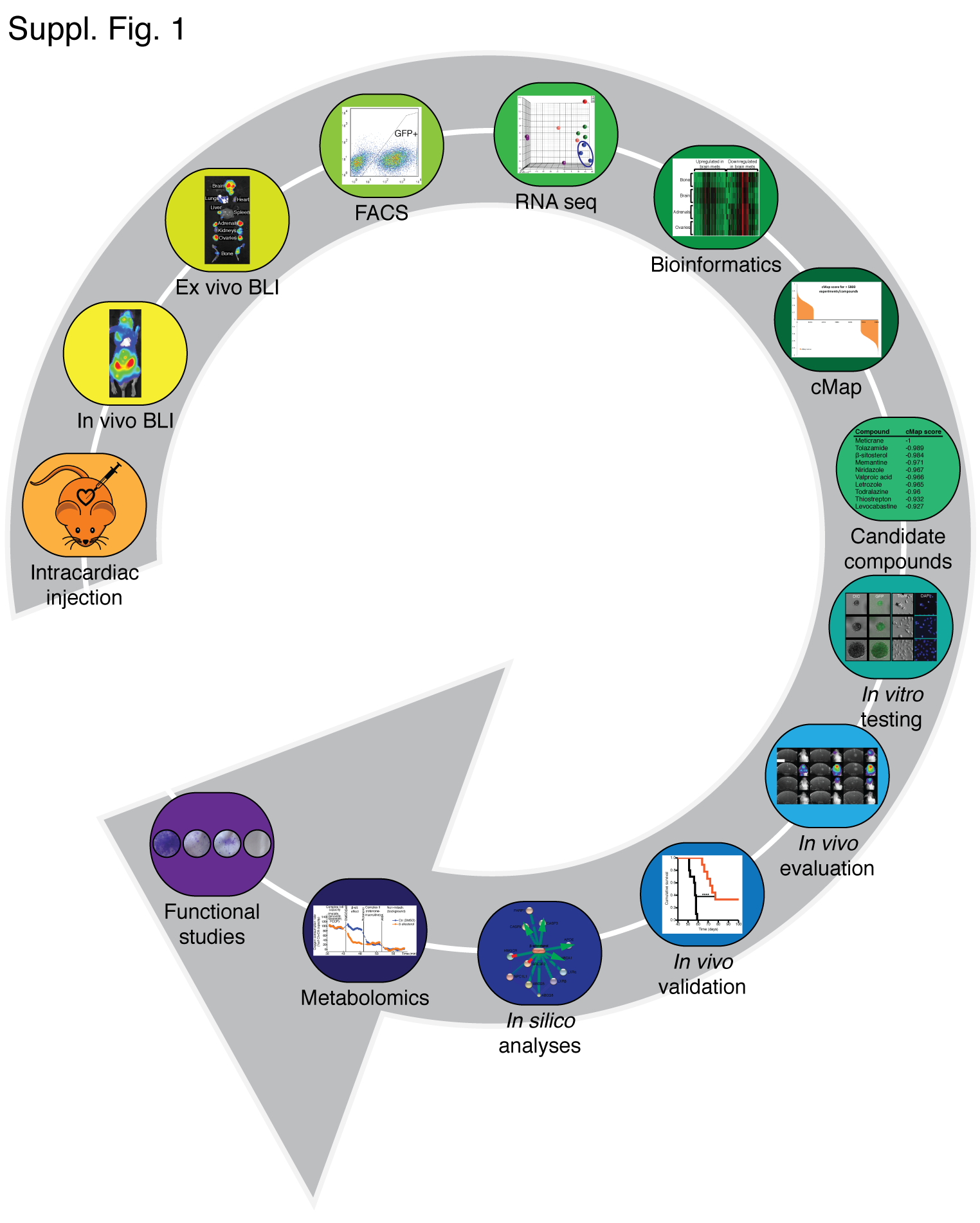

Supplement: Supplementary file 1 — Figure S1. The diagram illustrates the step-by-step workflow and analysis strategy used in the current study. (TIF 1390 kb) [file 40478_2019_712_MOESM1_ESM.tif]

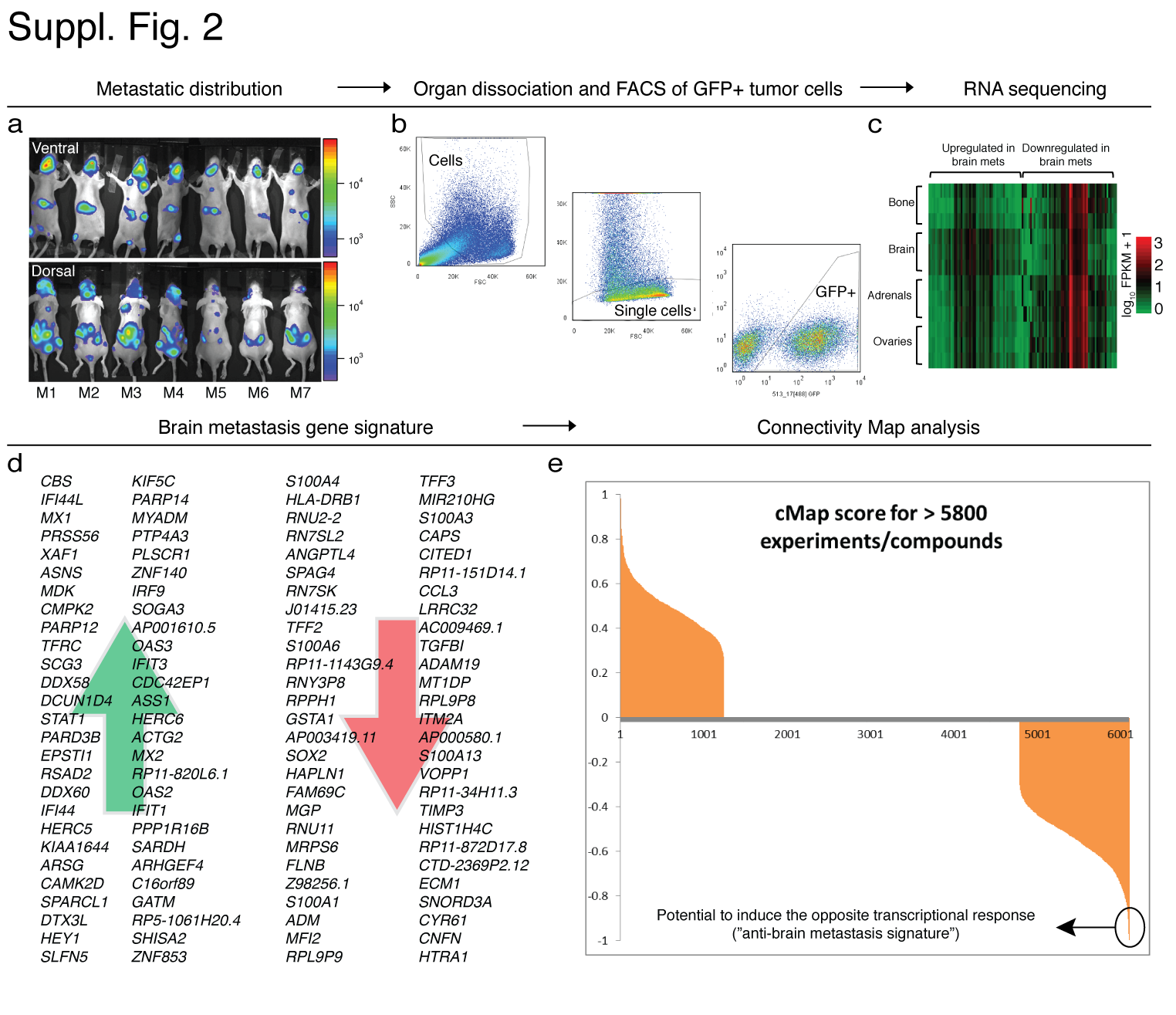

Supplement: Supplementary file 2 — Figure S2. Generation of organ samples for RNA sequencing, brain metastasis gene signature and Connectivity Map analysis. a BLI five weeks after intracardiac injection of 5 × 10^5^ H1_DL2 cells in NOD.CB17-Prkdcscid/NcrCrl mice (n = 7). M1-M7, indicate mouse 1 to 7. b Fluorescence-activated cell sorting (FACS) of GFP-positive tumor cells from the metastatic lesions. The sorted cells were analyzed by RNA-seq (n = 7). c Heatmap of the 108-gene brain metastasis signature with organ samples in rows and genes in columns. d The brain metastasis gene signature with the left two panels showing 54 upregulated genes in brain metastases and the right two panels showing 54 downregulated genes. e Query results from the Connectivity Map (cMap) database using the 108-gene signature. A score of < 0 to − 1 means net reversal of the signature (negative correlation) and a score of > 0 to + 1 means net induction of the signature (positive correlation). (TIF 2033 kb) [file 40478_2019_712_MOESM2_ESM.tif]

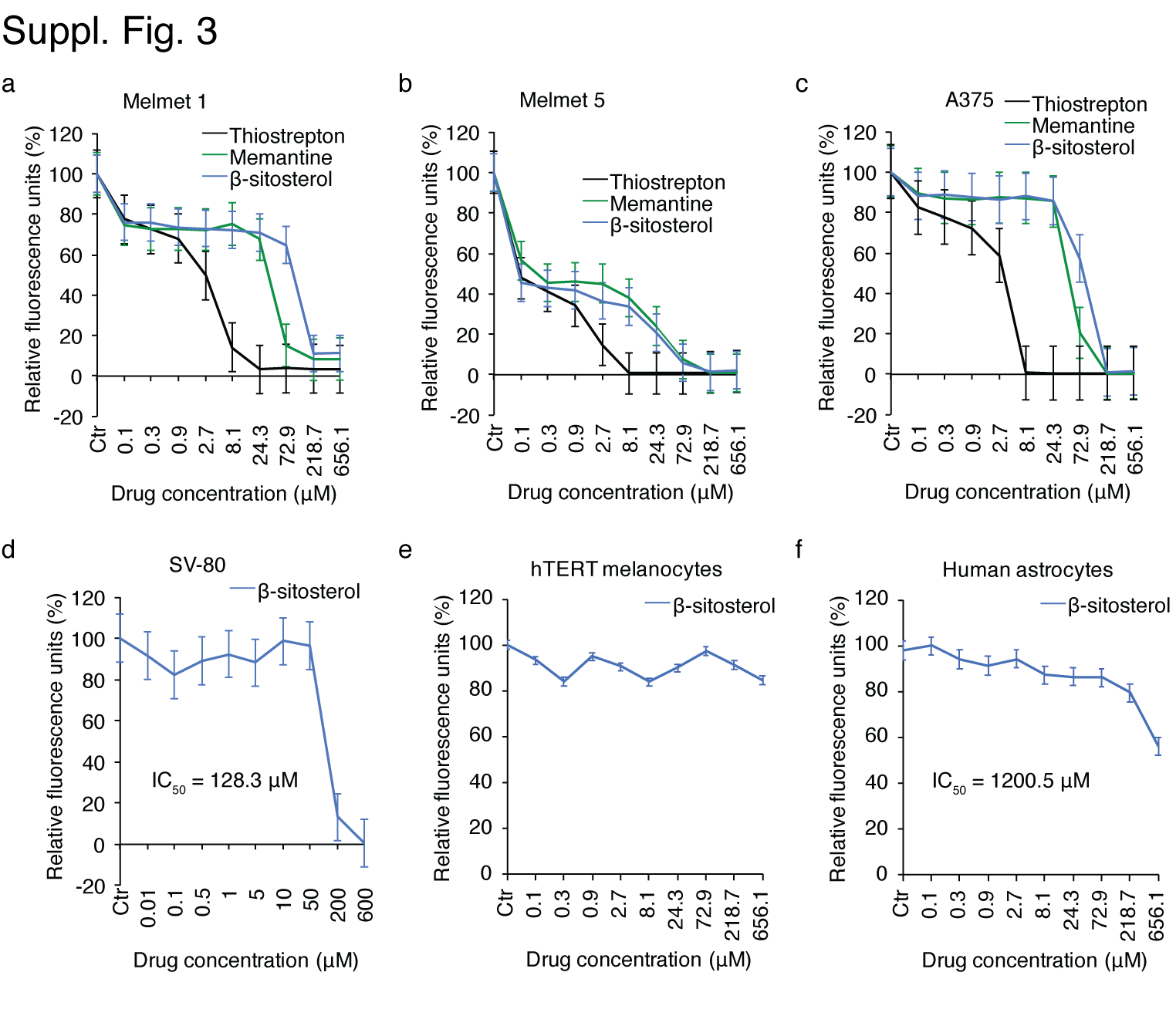

Supplement: Supplementary file 4 — Figure S3. Cell viability in vitro by increasing β-sitosterol concentrations. Human melanoma cell lines: a Melmet 1, b Melmet 5, and c A375 (n = 18 per cell line per drug per drug concentration). Normal cells: d SV-80 human lung fibroblasts (n = 18 per drug concentration), e hTERT immortalized melanocytes and f Human astrocytes. All values are given as the mean ± s.e.m. (TIF 943 kb) [file 40478_2019_712_MOESM4_ESM.tif]

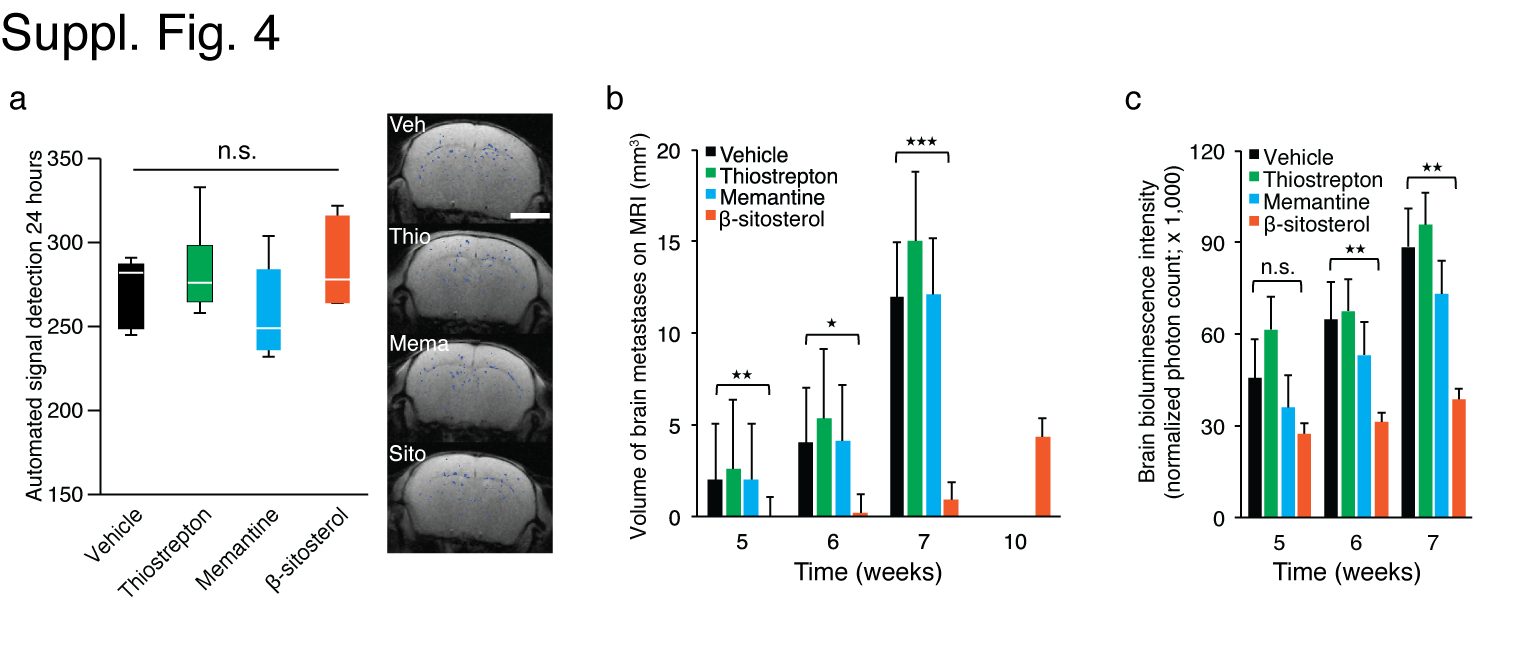

Supplement: Supplementary file 5 — Figure S4. In vivo efficacy of candidate compounds on H1_DL2 brain metastases. a Left panel: Tumor cell load into the brain was quantified by MRI-based automated quantification of nanoparticle-labeled cells in NOD/SCID mouse brains 24 h after intracardiac injection of 5 × 10^5^ H1_DL2 cells. Right panel: Images show typical MRI T2*-weighted images of mouse brains (scale bar, 0.25 cm) with an overlay of detected signals (blue). Study groups received 0.2 mL i.p. injections of 40 mg/kg thiostrepton every second day (n = 5), 10 mg/kg memantine daily (n = 5), 5 mg/kg β-sitosterol daily (n = 4) or vehicle (0.5% DMSO; n = 5) from one week post-injection. See Fig. 3 for more details on this experiment. b Volume of brain metastases determined by MRI (T1-weighted with contrast). Total volume of brain metastases per animal was compared at group level. c Brain BLI (total photon count = dorsal + ventral region of interest (ROI). a-c Student’s t-test: n.s. P ≥ 0.05, * P < 0.05, ** P < 0.01, *** P < 0.001. All values are given as the mean ± s.e.m. (TIF 3422 kb) [file 40478_2019_712_MOESM5_ESM.tif]

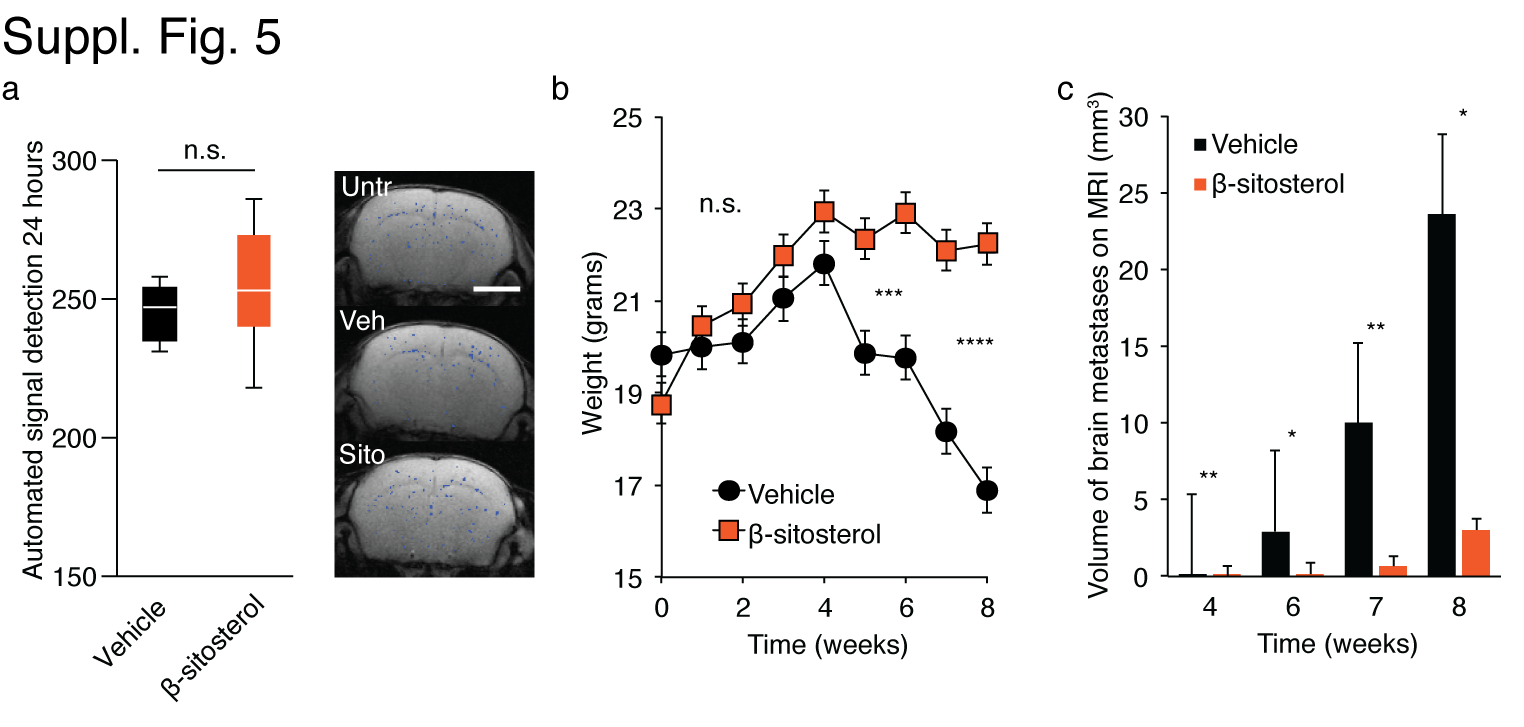

Supplement: Supplementary file 6 — Figure S5. In vivo validation of β-sitosterol on H1_DL2 brain metastasis. a Initial tumor cell load into the brain assessed by MRI-based automated quantification of nanoparticle-labeled cells 24 h after intracardiac injection of 5 × 10^5^ H1_DL2 cells. Images show typical MRI T2*-weighted images of mouse brains (scale bar, 0.25 cm) with an overlay of detected signals (blue). The study groups received daily i.p. injections of 0.1 mL vehicle (olive oil; n = 10) or β-sitosterol 5 mg/kg diluted in olive oil (n = 9) starting one week before tumor cell injections. See Fig. 4 for more details on this experiment. b Body weight from week zero to eight. At 100 days, the three remaining β-sitosterol mice had an average body weight of 26.7 ± 0.6 g. c Volume of brain metastases assessed by MRI (T1-weighted with contrast). Total volumes of brain metastases per animal were compared at group level. a-c Student’s t-test: n.s. P ≥ 0.05, * P < 0.05, ** P < 0.01, *** P < 0.001, **** P < 0.0001. All values are given as the mean ± s.e.m. (TIF 726 kb) [file 40478_2019_712_MOESM6_ESM.tif]

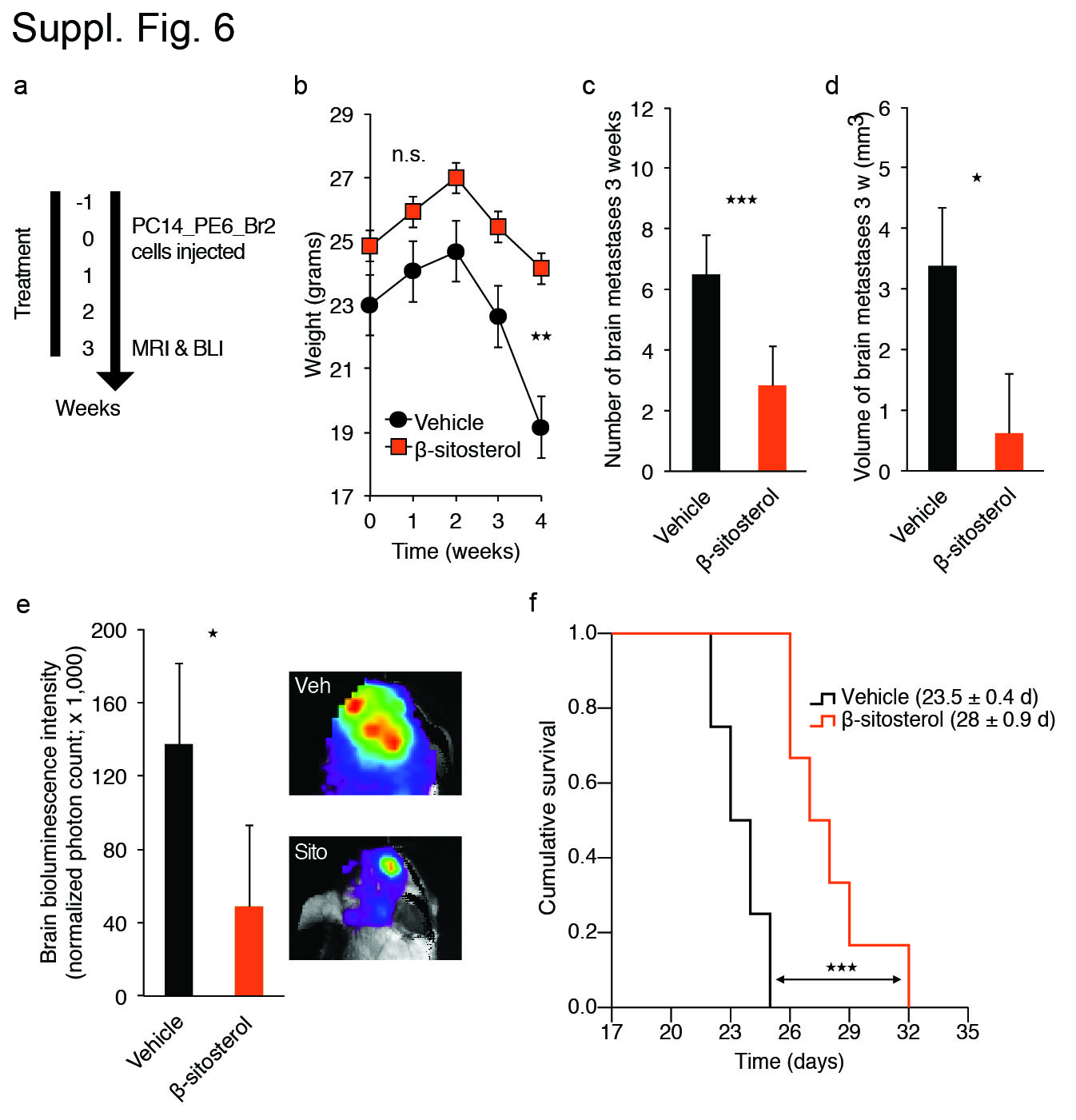

Supplement: Supplementary file 7 — Figure S6. β-sitosterol treatment effect is not model specific. a Experiment overview. Mice were injected intracardially with 5 × 10^5^ PC14_PE6_Br2 cells. Test groups received daily i.p. injections of 0.1 mL vehicle (olive oil; n = 4) or β-sitosterol 5 mg/kg (n = 6). Treatment started one week prior to tumor cell injections and was continued until euthanization. b Body weight from week zero to four for vehicle and β-sitosterol-treated mice. c Number and d volume of brain metastases at MRI at three weeks (T1-weighted with contrast). Total volume of brain metastases per animal compared at group level. e Brain BLI at three weeks (total photon count = dorsal + ventral region of interest). b-e Student’s t-test: n.s. P ≥ 0.05, * P < 0.05, ** P < 0.01, *** P < 0.001. f Kaplan-Meier survival plot (Mantel-Cox log-rank test: *** P < 0.001). All values are given as the mean ± s.e.m. (TIFF 2165 kb) [file 40478_2019_712_MOESM7_ESM.tiff]

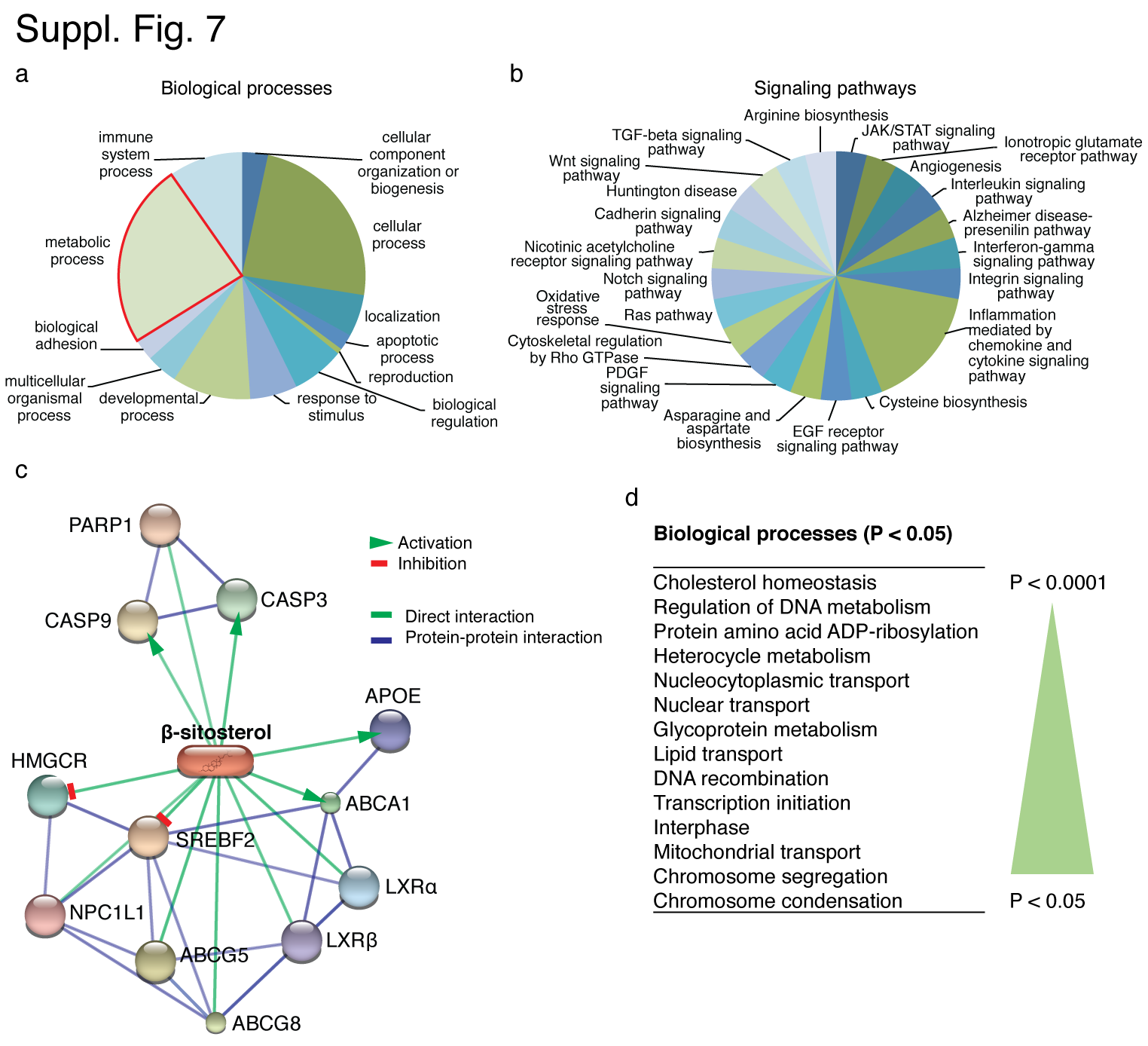

Supplement: Supplementary file 8 — Figure S7. a Functional classification of the brain metastasis gene signature. Human biological processes (metabolic processes are highlighted in red), and b signaling pathways associated with the 108-gene brain metastasis signature using the Protein Analysis Through Evolutionary Relationships (PANTHER) classification system. c Known and predicted protein interactions of β-sitosterol from the Search Tool for Interactions of Chemicals (STITCH 4.0). Green lines represent direct interactions with β-sitosterol whereas blue lines represent protein-protein interactions. Direct activation (green arrows) or inhibition (red lines) is indicated. d Significant statistical associations of β-sitosterol targets with cellular processes predicted with the Human Experimental/Functional Mapper (HEFalMp). P-values represent approximate multiple hypothesis corrected values. (TIF 1333 kb) [file 40478_2019_712_MOESM8_ESM.tif]

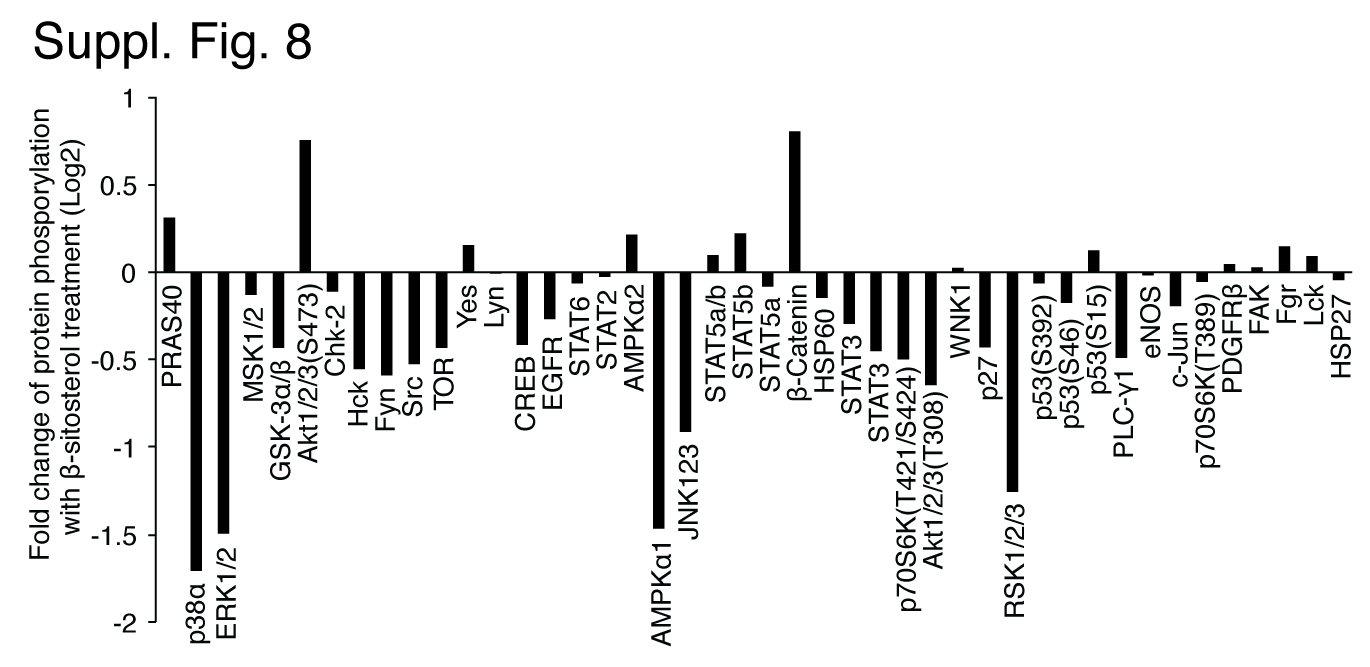

Supplement: Supplementary file 9 — Figure S8. Protein phosphorylation screening following β-sitosterol treatment. Parallel determination of protein phosphorylation levels (43 oncogenic kinases and two total proteins) in H1_DL2 cells after 24 h treatment with vehicle (0.05% DMSO) or β-sitosterol (50 μM). (TIF 418 kb) [file 40478_2019_712_MOESM9_ESM.tif]

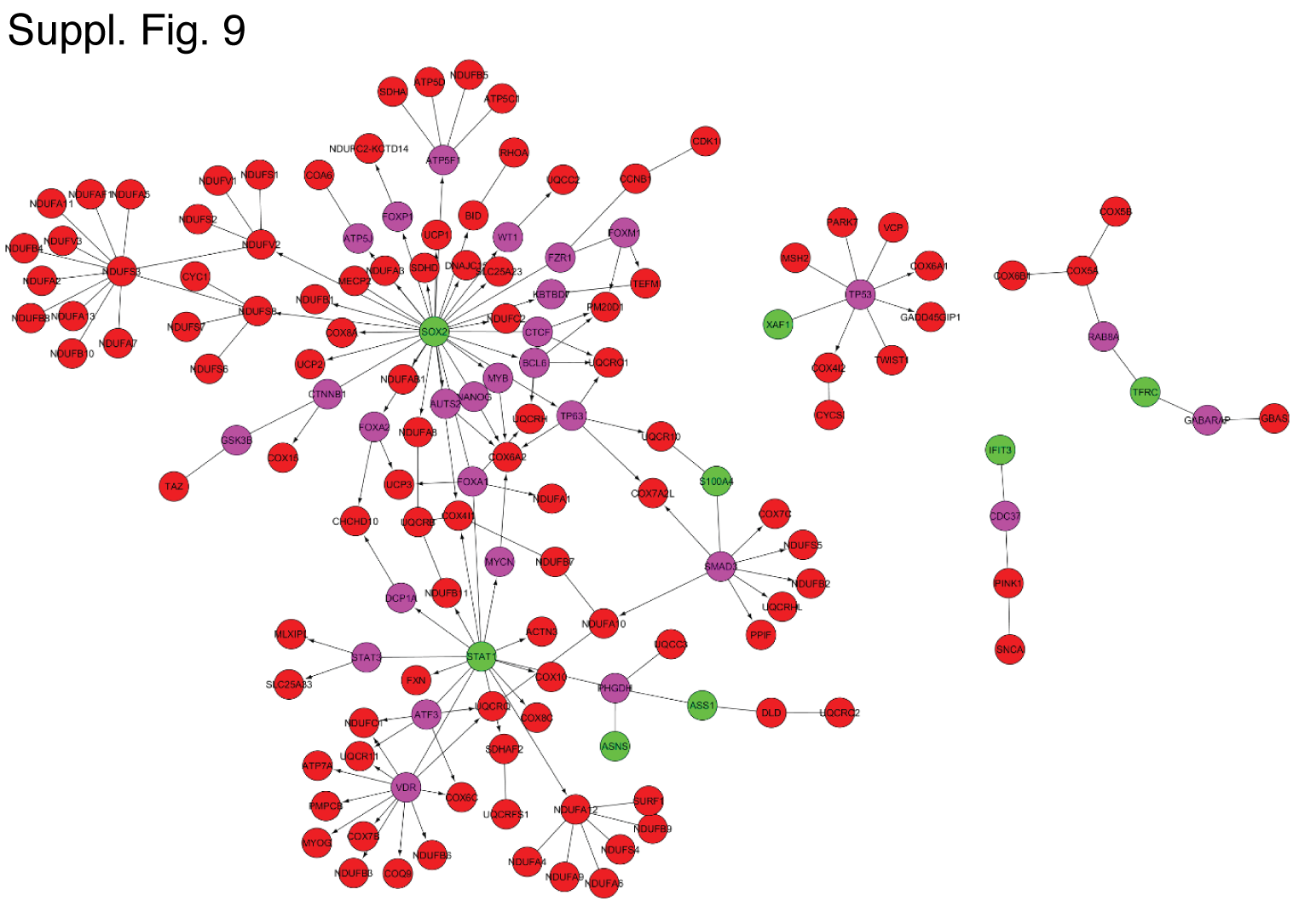

Supplement: Supplementary file 10 — Figure S9. Interaction network of signature genes and oxidative phosphorylation. The 121 Gene Ontology (GO)-annotated genes implicated in oxidative phosphorylation (red nodes) have 163 experimentally validated protein-protein and protein-DNA interactions involving 143 genes/proteins, including eight signature genes (green nodes). Purple nodes: Other genes that mediate interactions between signature and oxidative phosphorylation genes. Interaction directionality is represented with arrows. (TIF 1057 kb) [file 40478_2019_712_MOESM10_ESM.tif]

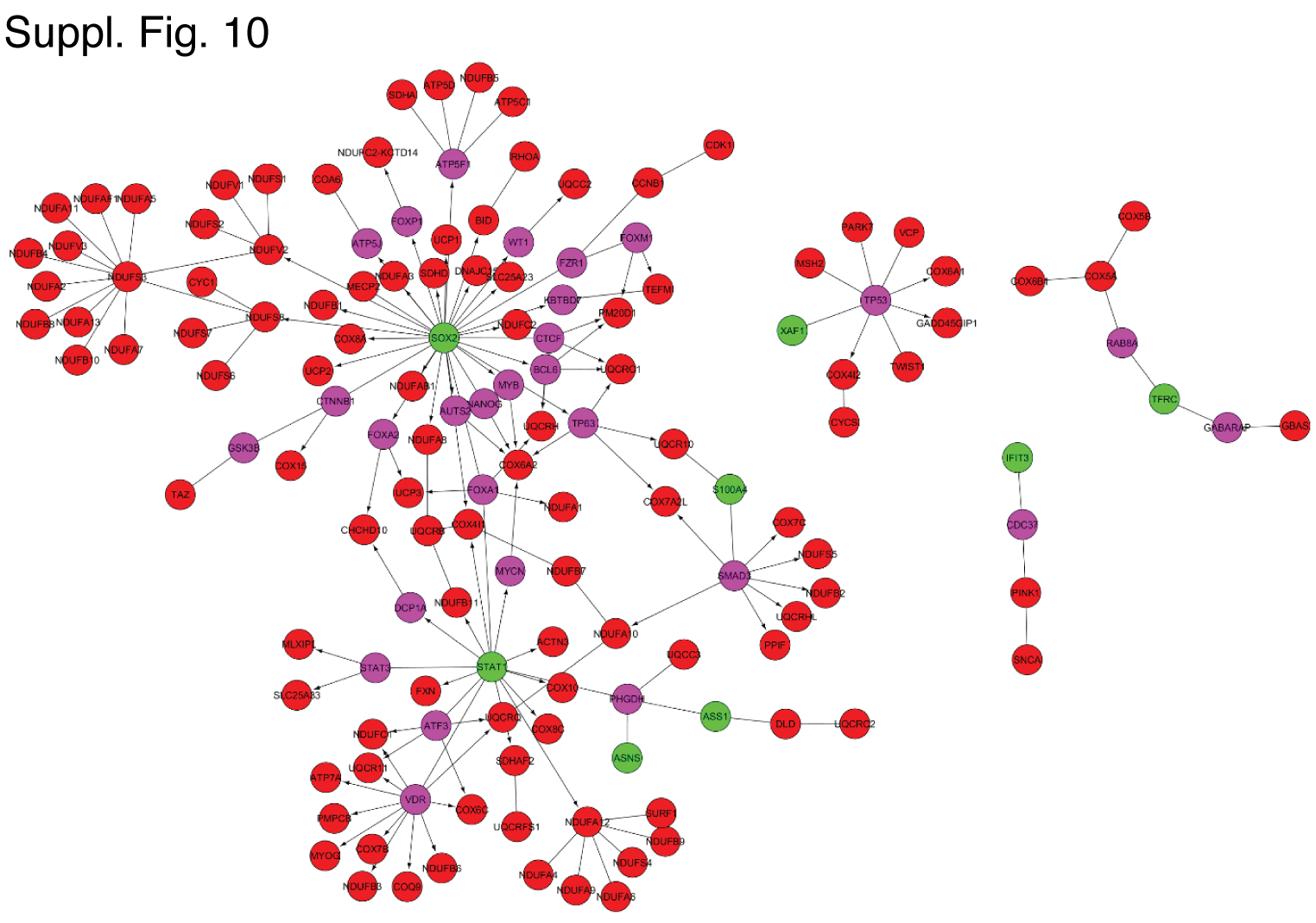

Supplement: Supplementary file 11 — Figure S10. Interaction network of β-sitosterol targets and oxidative phosphorylation. The 121 Gene Ontology (GO)-annotated genes implicated in oxidative phosphorylation (red nodes) have 155 experimentally validated protein-protein and protein-DNA interactions involving 141 genes/proteins, including four β-sitosterol targets (green nodes). Purple nodes: Other genes that mediate interactions between β-sitosterol targets and oxidative phosphorylation genes. Interaction directionality is represented with arrows. (TIF 1056 kb) [file 40478_2019_712_MOESM11_ESM.tif]

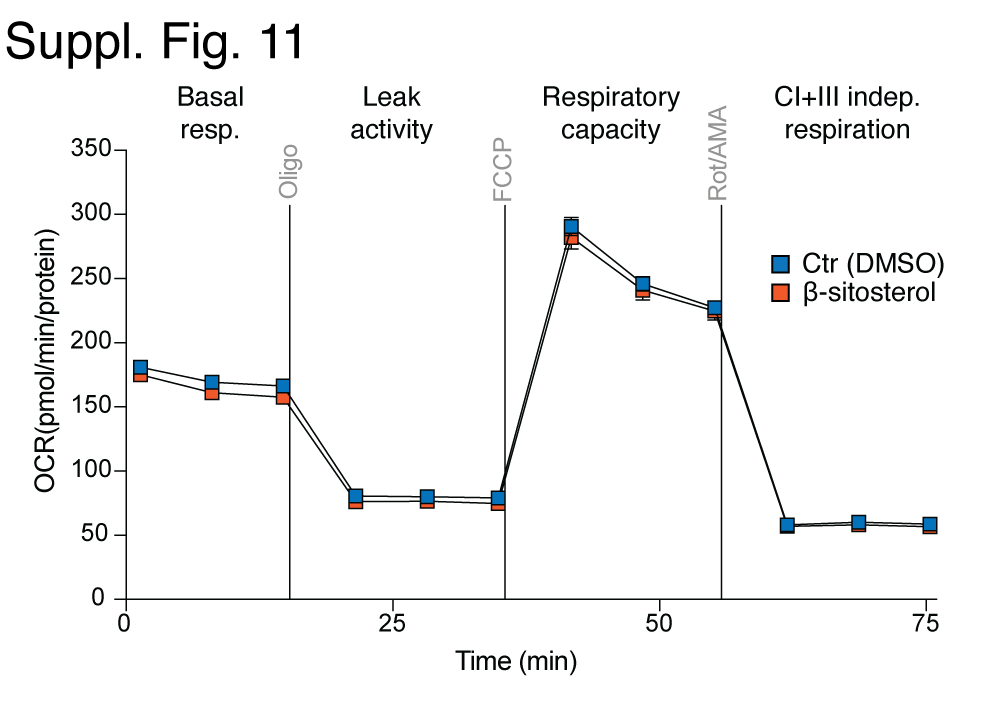

Supplement: Supplementary file 12 — Figure S11. The respiratory capacity (OCR) in normal melanocytes following β-sitosterol treatment as measured by the Seahorse system. Compared to the tumor cells (Fig. 5a) no changes in respiratory capacity was observed. (TIF 263 kb) [file 40478_2019_712_MOESM12_ESM.tif]

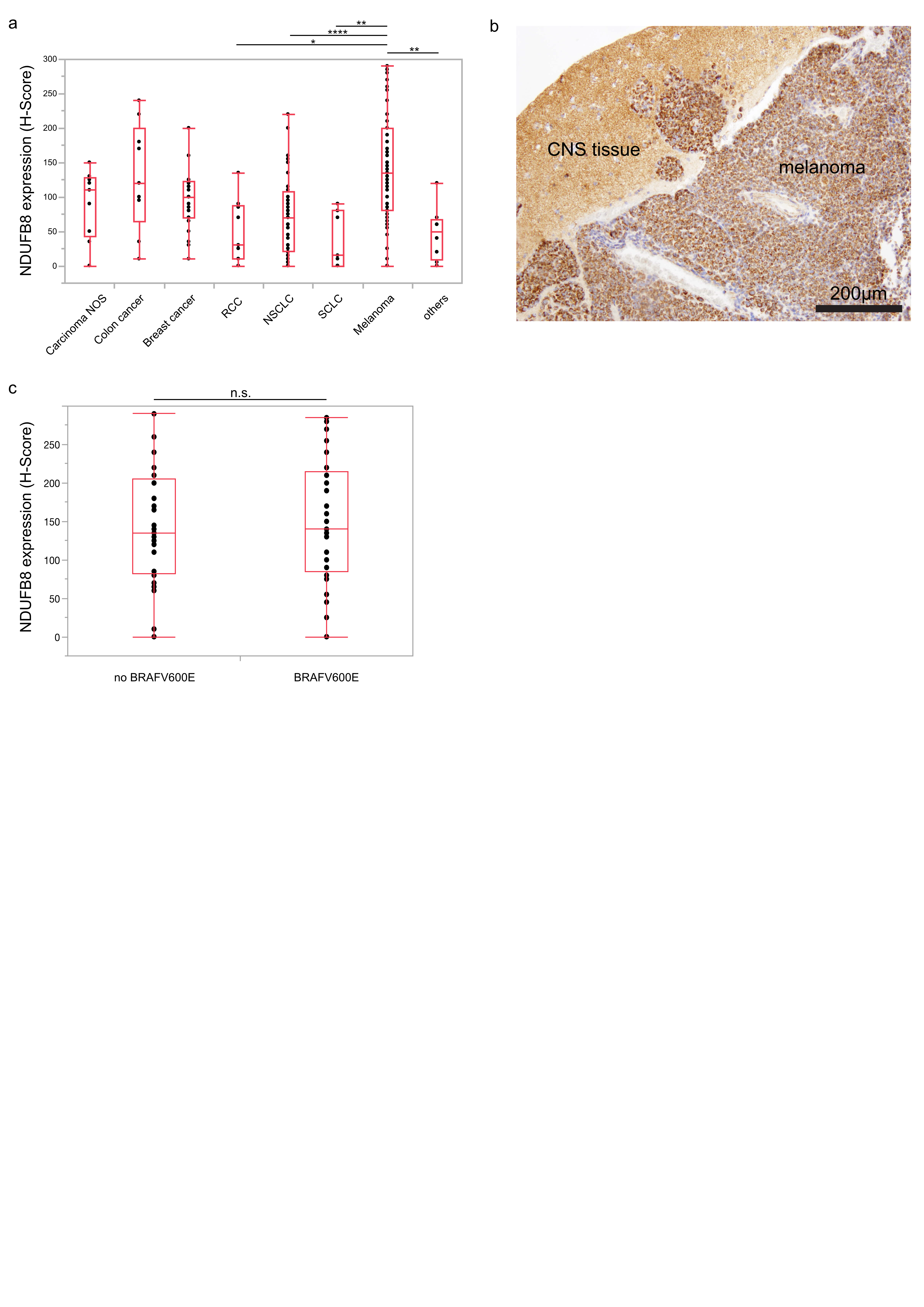

Supplement: Supplementary file 13 — Figure S12. Mitochondrial complex I activity in human brain metastases. a Expression levels of complex I subunit NDUFB8 in 197 human brain metastases from different cancers: Carcinoma not otherwise specified (NOS; n = 9), colon cancer (n = 9), breast cancer (n = 25), renal cell cancer (RCC; n = 9), non-small cell lung cancer (NSCLC; n = 52), small cell lung cancer (SCLC; n = 7), melanoma (n = 78) and others (n = 8). H-Score = [staining intensity (1 = weak, 2 = moderate, 3 = strong)] x [frequency of positive cells (%)]. Kruskal-Wallis test with Dunn’s multiple comparisons test: * P < 0.05, ** P < 0.01, **** P < 0.0001 and for comparisons not indicated P ≥ 0.05. b Immunohistochemistry of NDUFB8 in a human melanoma brain metastasis. c Expression levels of complex I subunit NDUFB8 in BRAF mutated melanoma (BRAFV600E; n = 24) and BRAF wild type melanoma (no BRAFV600E; n = 23). Kruskal-Wallis test with Dunn’s multiple comparisons test: n.s.: not significant. (TIFF 12141 kb) [file 40478_2019_712_MOESM13_ESM.tiff]
