# Supplementary material for: Inhibition of mitochondrial respiration prevents BRAF-mutant melanoma brain metastasis
Source: Acta Neuropathol Commun. 2019 Apr 10;7:55. doi: 10.1186/s40478-019-0712-8 (PMC6456988; doi:10.1186/s40478-019-0712-8)
Supplement: Supplementary file 3 — Table S1. Tissue Digestion Protocols. Preparation of Liberase TM Research Grade (Roche Applied Science) working solution and organ-tailored protocols for tissue digestion (brain, adrenals, ovaries and bone). (DOCX 18 kb) [file 40478_2019_712_MOESM3_ESM.docx]

**Prepare 30 mL Liberase TM Research Grade (Roche Applied Science) working solution:**

| 1. Thaw Liberase stock solution (13 U/mL) on ice. |
| --- |
| 1. Add 323 μL Liberase stock to 29.677 μL pre-warmed serum-free DMEM to prepare 0.14 U/mL working solution: (0.14 U/mL) (30 mL) = (x) (13 U/mL) |

**Brain, adrenals, and ovaries:**

| 1. Collect organ in sterile PBS-/- in a 35 mm petri dish and keep on ice until processing. |  |
| --- | --- |
| 1. In a 100 mm culture dish, cut into a fine puree using two scalpel blades (clean and disinfect scalpel blade with 70% EtOH prior to each use). |  |
| 1. Pipette or scrape the puree into a 15 mL falcon tube. |  |
| 1. Add 6 mL (for brain) or 5 mL (for adrenals and ovaries) of Liberase TM research grade digestion buffer (0.14 Wunsch units/mL; 26.87 μg/mL diluted in regular serum-free DMEM) to the organ puree. If organ tissue is small, especially adrenals and ovaries: Reduce amount of Liberase/medium accordingly. | |
| 1. Incubate at 37 ^°^C for 30 min with agitation every 5 min. | |
| 1. Pipette up and down gently with 1000 μL pipette. Try to break up clumps as much as possible. | |
| 1. Filter through a sterile nylon membrane (~50 μm mesh) into a 50 mL falcon tube and then transfer filtrate to a 15 mL tube (brain). Adrenals and ovaries can be filtered directly to a 15 mL falcon tube. | |
| 1. Add equal volume of cold DMEM complete medium with 10% FBS to the tube. |  |
| 1. Centrifuge 100 G × 1 min (brain) or 1200 rpm × 5 min (adrenals and ovaries) at 4 ^°^C to pellet cells. | |
| 1. Only brain: Remove supernatant and resuspend in 5 mL cold DMEM complete medium w/ 10% FBS. |  |
| 1. Only brain: Filter through a 35 μm membrane cap into another 15 mL falcon tube. |  |
| 1. Only brain: Centrifuge 100 G × 1 min at 4 ^°^C to pellet. | |
| 1. Remove supernatant and resuspend in 3 mL (brain) or 2 ml (adrenals and ovaries) cold ACK lysing buffer (full concentration). |  |
| 1. Incubate on ice 5 min. |  |
| 1. Add 8 mL cold DMEM complete medium w/ 10% FBS. |  |
| 1. Centrifuge 100 G × 1 min (brain) or 1200 rpm x 5 min (adrenals and ovaries) at 4 ^°^C to pellet. | |
| 1. Remove supernatant and resuspend in 500 μL cold DMEM complete medium w/ 1% FBS. | |
| 1. Filter through a 35 μm membrane cap into a 5 mL sterile polypropylene round-bottom tube. If worried there are few cells in a particular sample, either forego this final filtering step and add the cells directly to the final 5 mL tube, or filter the 500 μL of cells through the 35 μm membrane into the 5 mL collection tube, then wash the membrane with another 500 μL of medium. Centrifuge the collected cells at 1200 rpm × 5 min at 4 °C to pellet and then remove at least 500 μL of the supernatant. | |
| 1. Remove 20 μL of cell sample and mix w/ 20 μL Trypan Blue. Count viable cells with hemacytometer. Final concentration should be 1-2 × 10^7^ cells/mL. | |
| 1. Place on ice for cell sorting. |  |

**Bone:**

| 1. Collect tibia and femur from both legs, cut off as much muscle as possible before placing in sterile PBS-/- in a 35 mm petri dish, and keep on ice until processing. |  |
| --- | --- |
| 1. In a 100 mm culture dish, continue to remove muscle until only bones are left. |  |
| 1. Clean a mortar and pestle with 70% alcohol. |  |
| 1. Place the bones in the mortar, and add 200 μL of cold DMEM complete medium with 10% FBS. |  |
| 1. Grind the bones with the pestle for 2 min. Add 500 μL of cold DMEM complete medium with 10% FBS, resuspend in mortar. |  |
| 1. Filter the cell suspension through a sterile nylon membrane (~50 μm mesh) into a 15 mL falcon tube. |  |
| 1. Repeat grinding, adding growth medium, and filtering into falcon tube, 2 more times. |  |
| 1. Spin down 1500 rpm (400 g) for 5 min. 2. Remove supernatant and resuspend in 2 mL cold ACK lysing buffer (full concentration). |  |
| 1. Remove supernatant and resuspend in 2 mL cold ACK lysing buffer (full concentration). |  |
| 1. Incubate on ice 5 min. |  |
| 1. Add 8 mL cold DMEM complete medium w/ 10% FBS. |  |
| 1. Centrifuge 1500 rpm x 5 min at 4 °C to pellet. | |
| 1. Remove supernatant and resuspend in 500 μL cold DMEM complete medium w/ 1% FBS |  |
| 1. Filter through a 35 μm membrane cap into a 5 mL sterile polypropylene round-bottom tube. |  |
| 1. Remove 20 μL of cell sample and mix w/ 20 μL Trypan Blue. Count viable cells with hemacytometer. Final concentration should be 1-2 x 10^7^ cells/mL. |  |
| 1. Place on ice for cell sorting. |  |
